# Should We Stop Looking for a Better Scoring Algorithm for Handling Implicit Association Test Data? Test of the Role of Errors, Extreme Latencies Treatment, Scoring Formula, and Practice Trials on Reliability and Validity

**DOI:** 10.1371/journal.pone.0129601

**Published:** 2015-06-24

**Authors:** Juliette Richetin, Giulio Costantini, Marco Perugini, Felix Schönbrodt

**Affiliations:** 1 Department of Psychology, University of Milan-Bicocca, Milan, Italy; 2 Department of Psychology, Ludwig-Maximilians-Universität München, Munich, Germany; Universitat Wien, AUSTRIA

## Abstract

Since the development of D scores for the Implicit Association Test, few studies have examined whether there is a better scoring method. In this contribution, we tested the effect of four relevant parameters for IAT data that are the treatment of extreme latencies, the error treatment, the method for computing the IAT difference, and the distinction between practice and test critical trials. For some options of these different parameters, we included robust statistic methods that can provide viable alternative metrics to existing scoring algorithms, especially given the specificity of reaction time data. We thus elaborated 420 algorithms that result from the combination of all the different options and test the main effect of the four parameters with robust statistical analyses as well as their interaction with the type of IAT (i.e., with or without built-in penalty included in the IAT procedure). From the results, we can elaborate some recommendations. A treatment of extreme latencies is preferable but only if it consists in replacing rather than eliminating them. Errors contain important information and should not be discarded. The D score seems to be still a good way to compute the difference although the G score could be a good alternative, and finally it seems better to not compute the IAT difference separately for practice and test critical trials. From this recommendation, we propose to improve the traditional D scores with small yet effective modifications.

## Introduction

The Implicit Association Test [1] is a well-known measure that has been designed to assess implicit preferences such as implicit attitudes or implicit self-esteem. Its most widely used form consists of a computerized classification task of two target categories (e.g., black versus white faces) and two attribute categories (e.g., positive versus negative words) with a 7-block structure in its classic form. The logic underlying the IAT is based on response interference or compatibility. If one has an implicit preference for white over black individuals, it should be easier to classify positive words and white faces with a single key and negative words and black faces with another key (one critical block) than to classify negative words and white faces with the same key and positive words and black faces (another critical block). The easiness of the task is evaluated through reaction times and error rates and the IAT effect is defined as the difference between the two critical blocks.

Since the development of the IAT [1], researchers have conducted studies to test its psychometric properties. In these studies, they examined the IAT’s predictive validity (see [2,3] for reviews), convergent and discriminant validity (e.g., [4,5]), and reliability (e.g., [6,7]). However, since Greenwald et al. [8] identified the D score (difference between the critical blocks means divided by the inclusive *SD*) as the best way to compute the IAT score, most of these tests of psychometric validity of the IAT have been performed only using this particular scoring method. In other words, research has mainly considered the psychometric properties of one type of IAT score. Psychometrically speaking, when testing the properties of a measure, it is always the test of a score as the outcome of this measure. In fact, although one tends to make no distinction between the two, one tests the validity of the score and not the validity of the measure. In this perspective, we argue that the type of algorithms used for scoring the IAT effect is a very important issue.

There are two main approaches for converting IAT performance into a score. In the first one, one elaborates a score applying treatments or mathematical operations on the data considering different parameters that might be relevant for IAT data. The other one is dedicated to identify different processes underlying IAT performances by applying mathematical modeling work in order to disentangle construct-related components (see [9] for a review). With this second approach, one can consider the identified estimate of the construct-related component as an alternative IAT score. For example, with the diffusion model, Klauer et al. [10] decomposed the IAT effect into different process components and identified the construct-related one. In a similar perspective, Meissner et al. [11] proposed the ReAL model in order to disentangle how much in the IAT effect is due to associations versus recoding using a multinomial processing tree model on erroneous and correct responses identifying one component related to the evaluative associations of the target categories. However, these approaches present some non-negligible disadvantages. For example, the specific components in the diffusion model usually show low reliability. In the ReAL model, one considers only the error data. This restriction led researchers to modify the original IAT to obtain error rates that allow computation of the equations. In sum, although these models can shed new light on the underlying processes of the IAT and thus allow a better understanding of what is being measured, the approach initiated by Greenwald and colleagues [1,8] is the most widely used and probably the easiest to bring improvement in terms of IAT scoring methods.

### The D score and some alternatives

Initially, Greenwald et al. [8] tested different candidates for computing the IAT score taking into account several parameters. They first considered the way to compute the difference between the critical blocks using the mean of average latencies for each block, the median, the logarithm transformed mean, and finally the D score that is the mean divided by the *SD* of the two critical blocks. They also used various error treatments, criteria for respondent exclusion (i.e., outliers at the level of the sample), treatments of extreme latencies (i.e., outliers at the level of the participant), and the inclusion or exclusion of practice trials. For testing the different algorithms, they examined convergent validity through correlations with self-report measures, internal consistency through test-retest correlation, resistance to general response speed influence, sensitivity to known influences (i.e., group differences), resistance to undesired influence of order of critical blocks, and resistance to the effect of prior IAT experience. Among the candidates, the D_2_ (for built-in penalty IAT) and the D_5_ or D_6_ (for no built-in penalty IAT) scores showed the best performance on data collected from the web. Their particular computations allow coping with possible speed-accuracy trade-offs by including time penalty to error trials (in the procedure or in the computation). The division by the standard deviation also reduces the influence of the general response speed. Although these scores demonstrate satisfactory results over the years, we believe that there still is room for improvement. To our knowledge, very few publications explored this possibility.

Glashouwer et al. [12] tested eleven algorithms with a no built-in penalty IAT on data collected in laboratory settings. In addition to four algorithms proposed by Greenwald et al. [8] (i.e., D_5_ with penalty of 2*SD* for errors, D_6_ with penalty of 600ms for errors, and log mean on practice or on both practice and test trials), they included seven alternative algorithms. Glashouwer et al. [12] elaborated these alternatives to test different hypotheses such as the importance of including versus excluding errors or the influence of general response speed. They evaluated the algorithms on similar properties than the ones used by Greenwald et al.[8], but they also added predictive validity that is, in our opinion, a very important property. They showed that the D measures had generally the best performance. Moreover, according to their results the inclusion of error trials does not seem as crucial as Greenwald et al. [8] suggested. Glashouwer et al. [12] argued that because error trials can be due to different reasons such as responding too fast or a lack of attention, adding penalties could mean adding noise to the measure.

Greenwald et al. [8]and Glashouwer et al.’s [12] works are important for providing a solid scoring method. They compared the performance of certain algorithms based on the correlations with direct measures or behavioral measures. However, they did not examine systematically the effects of each of the different variations they included in each scoring method (i.e., the parameters of the scoring algorithms) in terms of validity or reliability. Therefore, their results do not allow firm conclusions on the systematic effects of different ways to handle IAT data. Although Nosek et al.’ s [13] work has the same limitations, we believe their approach and findings can provide valuable information in terms of the important parameters to consider when scoring the IAT performance. The authors examined various scoring methods for the Brief IAT, a variant of the IAT that consists only of critical blocks [14] and not for the IAT, however some elements can be applied to the logic of the IAT. They considered different ways to compute the difference between critical blocks using the mean of latencies, the mean of average reciprocals, the mean of log-transformed latencies, the D score, and the G score. The G score [15] or the Gaussian rank latency difference, is a scale invariant, non-parametric dominance measure. It is computed by first deriving the fractional ranks (percentiles) of the subjects’ response latencies in the two response critical blocks and then by calculating the difference between the means of the Gaussian rank latencies in the two critical blocks (see [13] for the details on the computation). By considering the rank of the latencies rather than the raw latencies, it drastically reduces the influence of outliers in the distribution. The G score is very similar to the Brunner-Munzel Probability method, BMP [16], a rank-based method that estimates the probability that a RT from a critical block is faster than a RT from the other critical block. Compared to the BMP, the G algorithm includes a transformation in a comparable metric applied for computing the D score, resulting in an easier interpretation for those who are used to the D scores. Nosek et al. [13] also considered different ways to handle errors and extreme latencies. Examining several psychometric properties (e.g., internal consistency, convergent validity with direct measures), the G and D appeared to perform better than the other scoring methods compared to the simple mean, the median, or the mean of log-transformed latencies. Moreover, the performance of the G algorithm, compared to the Ds, has the advantage of being less dependent on removing or not the outliers in the initial distribution. In fact, G is an algorithm that includes robust statistics methods.

### Can robust statistics bring robustness to the IAT score?

Modern robust statistic methods can provide viable alternatives to existing scoring algorithms, especially considering the specificity of reaction time data. First, robust statistics are immune to non-normal distribution and lack of homogeneity of variance, the two main threats to classic parametric methods. Second, there is often a violation of these two conditions in RTs data. Logarithm transformations of reaction times are supposed to reduce skewness but sometimes fail to restore normality [17], and compress some information [18]. Therefore, the use of robust statistics in computing the difference between blocks might be beneficial in terms of validity. The good performance of the G score supports this idea.

Moreover, robust statistics offer a systematic way to deal with outliers as an alternative to previous methods at the sample and the individual levels. At the sample level, for the IAT or the BIAT, removing or recoding latencies is often considered (e.g., [8,13]). Usually, one would remove latencies above 10000ms and below 400ms. Alternatively, one would recode latencies below 300ms into 300ms and latencies above 3000 into 3000ms, respectively [8]. The cut-off points in the upper and lower tails are not set according to the distribution but to theoretical considerations. As a common rule, RTs below 300ms in a classification task are indicators of responses given without full information processing whereas RTs above 3000ms or 10000ms are indicators of distracted responses. At the individual level, in the D score, dividing the difference by the *SD* computed on both compatible and incompatible trials is a way to handle the heavy tails of the distributions affecting both means and *SD* (see [18], for a more detailed explanation). We believe that applying robust statistic methods would allow researchers to deal more systematically with outliers, at least at the individual level [19,20], and it could result in an improved scoring of the IAT. For example, Krause et al. [7], pointed out that trimming raw latencies before computing the scores for the Affective Priming Task [21] and the Identification-Extrinsic Affective Simon Task [22] increased the reliability of the scores from .55 to .67 and from .51 to .64, respectively (first occasion of measurement, Table 1, p. 245).

**Table 1:** Parameters and options under consideration for computing the tested algorithms.

**Parameter 1. Extreme Correct Latencies Treatment**	*Option 1*: No Treatment	*Option 2*: Fixed Values trimming (Eliminate latencies < 400 ms)	*Option 3*: Fixed Values winsorizing (Recode latencies < 300 & >3000ms)	*Option 4*: 10% Trimming (for each critical block, remove the 10% fastest and the 10% slowest latencies)	*Option 5*: 10% Winsorizing (for each critical block of the IAT, replace the 10% fastest latencies and the 10% slowest latencies)		*Option 6*: 10% Inverse Trimming (for each critical block of the IAT, eliminate the 10% of the latencies above and below the median latency)
**Parameter 2. Error Treatment**	*Option 1*: Ignore (no distinction between correct and error latencies when applying Parameter 1)	*Option 2*: Exclude (eliminate error latencies)	*Option 3*: Recode 2SD (replace error latencies with correct latencies mean RT + 2SD)	*Option 4*: Separate (applying Parameter 1 on extreme error latencies and extreme correct latencies separately)	*Option 5*: Recode 600 (replace error latencies with correct latencies mean RT + 600)		
**Parameter 3. IAT Score Formula**	*Option 1*: D (difference between the average latencies of the two critical blocks divided by the SD of all the latencies)	*Option 2*: G (procedure for computing the G scores is described in [15], Table 9)	*Option 3*: Worst Performance Rule (difference between the 90^th^ percentiles of the two critical blocks divided by the SD of all the latencies in both blocks)	*Option 4*: Mini difference (mean of all possible differences between the latencies of the two critical blocks divided by their SD)	*Option 5*: 10% trimming Mini Difference (option 4 but mean computed on the 10% trimmed differences)	*Option 6*: 10% winsorizing Mini Difference (option 4 but mean computed on the 10% winsorized differences)	*Option 7*: 10% Inverse trimming Mini Difference (option 4 but mean computed on the 10% inverse trimmed differences)
**Parameter 4. Distinction between practice and test critical blocks**	*Option 1*: No Distinction (score computed on practice and test critical trials together)			*Option 2*: Distinction (score computed separately for practice and test critical trials).			

The D_2_ is obtained following [8], by setting option 2 for Parameter 1 (fixed value trimming), option 1 for Parameter 2 (ignore), option 1 for Parameter 3 (D scores) and option 2 for Parameter 4 (distinction). The D_5_ and the D_6_ are obtained by setting option 3 (Recode errors latencies with *M* + 2*SD*) or 5 (Recode errors latencies with *M* + 600), respectively, with the other parameters being the same as for the D_2_.

### Aims of the contribution

Because the validity of the score is what determines the validity of a measure, the algorithm one uses for scoring the IAT effect matters. In this perspective, we aimed at identifying whether some ways to handle RT and error IAT data lead to better results in terms of different psychometric properties (reliability and validity—convergence with indirect and direct measures, and predictive validity). Although previous research allows establishing reliable recommendations on the scoring method, no study investigated systematically and statistically whether some ways were better than others. With this contribution, we aim at filling this gap. We applied the transformations on data from two types of IAT (i.e., built-in penalty procedure and no built-in penalty procedure) assessing different objects from self-esteem to attitude toward fruit versus dessert. By considering different domains and different IATs, we aimed at obtaining generalizable results across domains and across IAT procedures. If it is possible to identify one or more transformations that affect IAT scores, they should do so in a consistent manner. They should improve psychometric properties across domains. We crossed different options of four parameters considering several robust and non-robust statistics methods. We operationalized different ways of dealing with extreme latencies and ways of handling errors, various methods for computing the difference between the two critical blocks. Finally, we considered whether computing the IAT score with or without distinguishing between practice and test trials. In the method section, we explained and described in detail the four parameters and their options as well as the computational aspects of the algorithms. We tested the effects of the four parameters with robust ANOVA as well as their interaction with the type of procedure used for the IAT (i.e., built-in versus no built-in penalty). Based on these results, we formulated a series of recommendations for elaborating more robust and valid IAT scores. Finally, we discussed the importance of some parameters when assessing implicit preferences with the IAT. Although our contribution is not intended to shed light on specific processes underlying IAT performance, investigating the effect of some ways to handle the data might point out some elements that are more central than others when it comes to the validity of the IAT score.

## General Method

### Data Sets and IAT details

The first three datasets came from a very large online collection, “Attitudes 3.0” [23], that occurred from November 6, 2007 to May 30, 2008. There are three sets of data on three different domains: Political (Democrats vs. Republicans), Race (White vs. Black), and Self-esteem. For all three, the procedure included taking the same indirect measures but direct and behavioral measures applied specifically to each domain, resulting in a different number of indicators for each data set. The number of cases or participants depends on the criteria under consideration varying from 93 to 3003 for reliability indicators and from 288 to 675 for validity indicators (see Tables A-C in S1 File for details) with a mean age of 29.1 years’ old (*SD* = 12). For all three domains, the IAT procedure was the one used by Nosek et al. [24]. There was a 7-block structure with practice blocks of 20 or 40 (for switching single sorting block) trials and test blocks of 40 trials, resulting in 60 trials for each critical block for computing the IAT score. Error feedback was given and participants had to give the correct answer in order to continue to the next trial. The recorded reaction time included the time for making that correction (built-in penalty procedure). Finally, there was a between-participants randomization of the order of the completion of the critical blocks.

A second group of three datasets originated from three separate published studies, one data set on Fruit and Snack ([25], Study 2), one data set on Dessert and Fruit [26], and one data set on Morality ([27], Study 2). Data were collected in laboratory settings. For all three data sets, there were some different direct and behavioral measures with sample sizes around 100 (see details in Table D in S1 File) constituted mainly of students with mean ages of 25.1 (*SD* = 6.8), 23.2 (*SD* = 5), and 23.3 (*SD* = 4.6), respectively. The IAT had always the same 7-block structure with practice blocks of 20 trials and test blocks of 40 trials, resulting in 60 trials for each critical block for computing the IAT score. Error feedback was provided but participants did not have to give the correct answer to continue to the next trial thus the reaction time did not include any penalty for errors (no built-in penalty procedure). The order of the completion of the critical blocks was randomized between participants. Please refer to the three publications for more details.

#### Ethics Statement

The contribution is based on secondary data analysis. For each of the data sets, the authors of the original data collection obtained ethic approval.

### Psychometric properties under consideration

We examined two main psychometric properties that are reliability and validity. For establishing validity, we considered convergent validity with direct and indirect measures, and predictive validity (sample sizes used for computing each criterion, as well as a detailed list of the measures used in each dataset are presented in Tables A-D in S1 File).

#### Reliability

Reliability is an important but limited psychometric property on its own, given that it is a necessary but not sufficient condition for validity. Some variations of the IAT scores might reflect better the reliable variance due to method factors than others do, and therefore show increased reliability. However, maximizing reliability in spite of validity is not desirable. For the three datasets with built-in penalty, for which several participants completed the IAT more than once, we considered both Split-half reliability and Test-retest correlation, whereas for the other three datasets we considered only Split-half reliability.

#### Convergent/discriminant validity with direct measures

Convergent/discriminant validity with direct measures is important but it is theoretically dependent. If one advocates that direct and indirect measures tap into two different constructs, a modest correlation between the IAT score and the direct measures is desirable. On the other hand, if one advocates that the two measures are tapping into the same construct one would expect the correlation to be more important (see [12] for a discussion). In order to compare the algorithms as done in previous research on the score of the IAT and its variants (e.g., [8,13]), we decided to put aside the theoretical perspective and concluded that pragmatically higher correlations between the IAT score and measures of explicit attitudes were desirable considering it more as an indicator of convergent rather than discriminant validity. We considered the correlations between the tested algorithms and four (Political and Race data), six (Self-Esteem data), or one (Fruit/Snack, Dessert/Fruit, and Morality) direct measures.

#### Convergent validity with other indirect measures

Concerning convergent validity of the IAT score with indirect measures, higher correlations were desirable. For estimating that criterion, we used the correlations with seven indirect measures (e.g., Go/No go Association task, Affect Misattribution Procedure, Evaluative Priming; see Appendix for the full list of measures) for each of the datasets with built-in penalty (Political, Race, and Self IAT) and one for the Dessert/Fruit data set, while no indirect measure other than the IAT was available in the Fruit/Snack and in the Morality datasets.

#### Predictive validity

This is an important psychometric property to consider when evaluating a measure or a scoring method, as one of the main goals of a measure is to predict outcomes. Two criteria were available for Political data, one criterion for Race data, two for the Fruit/Snack (including a correlation with an objective behavioral measure) and the Dessert/Fruit data sets, and one for the Morality data set (correlation with an objective behavioral measure).

### Parameters under consideration for handling IAT data

We computed a series of algorithms considering the combinations of four parameters: Parameter 1 (Extreme correct latencies Treatment), Parameter 2 (Error latencies treatment), Parameter 3 (IAT scores formula), Parameter 4 (Distinction between practice and test critical blocks) (see Table 1 for a summary of the different options of the four Parameters).

#### Parameter 1: Extreme Latencies Treatment

Because outliers in reaction measures are one of the main issue for RT data, we considered whether and how to handle them with six possible options. To prevent very extreme latencies from influencing the scores we always excluded correct and error latencies longer than 10s. The first option No Treatment serves both as a baseline and was suggested in [8]. Options 2 and 3 refer to fixed value outlier treatment: The cut-off points in the upper and lower tails are set according to theoretical considerations commonly used in the treatment of RT data for the IAT (see Introduction). Option 2, Fixed values Trimming, includes eliminating the latencies of the lower and upper tails at predetermined cut-off points. We only removed latencies below 400ms because we applied the upper tail treatment (latencies above 10000ms) for all algorithms, following Greenwald et al.’s [8] recommendations. Option 3, Fixed values Winsorizing, corresponds to replacing the trimmed scores to the predetermined cut-off boundaries: Latencies below 300ms were recoded to 300ms and latencies above 3000ms were recoded to 3000ms as suggested in [1] and as recommended in [8]. Options 4, 5, and 6 refer to a statistical outlier treatment separately for the two critical blocks of the IAT: The cut-off points in the upper and lower tails are set according to the empirical RT distribution. Among the different ways to handle outliers for location measures in the robust statistics literature, we opted for statistical trimming and winsorizing as they are the most common methods (see [19] for a review of the different methods). We chose 10% for the cut-off points (i.e., removing or replacing the 20% of latencies) because it should allow to get rid of extreme latencies without losing too much information. Whereas the option 4, 10% Statistical Trimming, corresponds to cutting symmetrically the lower and upper tails, the option 5, 10% Statistical Winsorizing, replaces the trimmed latencies by the last untrimmed latencies resulting in preserving the information that a case was in the upper or lower tail [28]. The option 6, 10% statistical Inverse Trimming, corresponds to cutting symmetrically around the mean instead of the lower and upper tails. It could be a valid alternative: Classic statistical trimming or winsorizing might remove important information because the IAT effect could reside in the tails of the distribution (i.e., faster or slower latencies than average). It is worth noting that inverse-trimming is not robust against outliers and therefore it cannot be considered a robust statistic [19]. Note that in general we applied the extreme latencies treatment on the correct latencies with two exceptions specified in the next parameter.

#### Parameter 2: Error Treatment

We considered five different options about how to treat error latencies because from previous work it appears that the way to treat errors is still is an unresolved issue [8,12]. Applying different error treatments for both types of IAT data (from built-in vs. no built-in procedure) in the same contribution might help to clarify whether and how to consider error latencies. We thus chose to include the type of error treatment as a parameter for datasets with and without the built-in penalty. The type of error treatment is intertwined logically with the extreme latencies treatment (Parameter 1). In the option 1, Ignore, there is no distinction between error and correct responses such that we applied the treatments of outliers (Parameter 1) on the distributions regardless of the correctness of the response. Therefore, this option has different implications if the IAT includes a built-in penalty procedure, in which the error latencies already include a penalty, and in the IAT without the built-in penalty, in which error latencies receive no penalty. In the option 2, Exclude, we simply removed the error latencies from the distribution and thus applying extreme latencies treatment only on correct responses. This option serves as a baseline that allows testing whether considering error latencies can make a substantial difference. Option 4, Separate, implies that we applied the extreme latencies treatment separately on error and correct latencies. The built-in penalty procedure results in error latencies on average much longer than correct ones because they include the necessary time for giving the correct answer. Therefore, treating them together with correct latencies leads to the selective exclusion or replacement of error latencies in the slower tail of the distribution (for trimming and winsorizing, respectively) or in the selective exclusion of correct latencies in the center of the distribution (for inverse trimming). Moreover, if one considers the status of the answer rather than the procedure itself, applying transformations separately for error and correct latencies can also make sense for no built-in penalty procedure IAT. The options 3 and 6 imply adding a fixed penalty to error latencies for each critical block, as proposed by Greenwald et al. [8]. Because the built-in penalty procedure might increase the proportion of construct-unrelated variance (i.e., the variability in term of general response speed), we chose to apply the Recode option for both no built-in and built-in penalty procedure IATs. We considered the two error recoding techniques initially used by Greenwald et al. [8]. Error latencies are replaced within each critical block by the block mean plus a penalty of 600ms (option 3) or by the block mean plus a penalty of 2 standard deviations (option5).

#### Parameter 3. IAT Score Formula

We considered seven possible ways to compute the IAT scores. Among them, we chose not to include the simple mean, median, or log-transformed mean because in previous studies the D score always over-performed all these options [8,13]. The standard D, option 1, corresponds to computing the difference between the means of the latencies in the two critical blocks divided by the pooled *SD*. This modified effect size has been proposed by [8] and has become the standard procedure to score IAT data. The G score, option 2, has been originally proposed for scoring the Brief-IAT [13] and is in fact a rank-based robust statistics procedure^1^. Ratcliff et al. [29] proposed the Worst Performance Rule (WPR) based on the results they obtained for the drift rate in the diffusion model: The slowest quantile (.90) of a RT task correlated more strongly with the drift rate than did the fastest quantile. We included the difference between the slowest quantiles (.90) divided by the pooled *SD* to actually test whether the worst performance rule would improve the psychometric properties of the IAT scores (option 3). We also included a new way to compute the difference we called Mini Differences (option 4). The idea behind the IAT score is to reveal a difference in terms of reaction time when one uses the same or a different key to categorize stimuli from a given target category (i.e., fruit) and stimuli from an attribute category (i.e., positive). In the D score, we assessed this difference with one single indicator obtained from the difference between the means from the two critical blocks. However, in terms of measurement of a concept, the more items or indicators one uses, the more reliable the score is. The Mini Differences scores allows taking into account this perspective and considers all possible differences between RTs from incompatible block and RTs from compatible block. We called them mini differences and then averaged them to obtain an IAT score. For example, if one considers all 60 trials in each critical block this procedure results in 3600 difference indicators. We then computed the average of these differences, divided by their standard deviation. This simple score is intended as a baseline for additional options, depending on how outliers and errors are handled in the differences. The use of the mini-differences allows to perform trimming (option 5), winsorizing (option 6), and inverse-trimming (option 7) on the distribution of the mini differences.

#### Parameter 4

Distinction between practice and test critical blocks. Usually in the IAT, there are two types of trials for the critical blocks, the practice and the test trials. The most common partition would be 20 practice trials and 40 critical trials. Greenwald et al. [8] suggested computing the IAT scores separately for practice and test trials and then calculating the final score as the mean between the two scores. However, this type of computation gives more weight to practice trials whereas they are fewer. We believe considering all trials together without weighting could reflect better the implicit measured concept and thus lead to a more valid score. In order to test whether it is an important factor to consider practice and test trials separately or not when computing the critical block difference, we included the two options (option 1: No distinction vs. option 2: Distinction).

### Implementation and planned analyses

Using all the possible combinations of the parameters, we implemented 420 algorithms (6 x 5 x 7 x 2) in the R package *IATscores* (see S3 File for where to find it and how to use it). However, options 1 and 2 for Error Treatment (i.e., Parameter 2: Separate and Ignore, see Table 1) produced identical results when the Extreme correct latencies Treatment (Parameter 1) did not entail thresholds that relied on the distribution of the latencies (options 1, 2, or 3, see Table 1). We considered this dependency among the parameters in the analyses: For all the analyses that did not involve error treatment as the independent variable, we considered the duplicate algorithms only once, resulting in 368 unique scoring algorithms. In the analyses involving Parameter 2 as an independent variable, we included the duplicates for the purpose of a fair comparison between options 1 and 2 on the one hand and the other options on the other, but we performed also a direct comparison of the options 1 and 2 after removing all the duplicates. The most used D scores (i.e., D_2_ for built-in penalty data and D_5_, and D_6_ for no built-in penalty data) as well as the G score as defined by [13] were included in the set of algorithms as the results of the combination of certain options of the different parameters.

We aimed at identifying which options for each parameter resulted in best performances. To do so, for each algorithm and for each dataset, we calculated indicators of the psychometric properties discussed above: Reliability, convergent validity with indirect measures, convergent validity with direct measures, and predictive validity. We computed the score for each property as the rank score of the algorithm, the highest rank corresponding to the best performance. When more than one indicator was available, we considered the average of the rank scores. Finally, we considered on the one hand a score of reliability and on the other hand a score of overall criterion validity within each dataset computed by averaging convergent validity with direct measures, convergent validity with indirect measures, and predictive validity.

For each of the four parameters separately, we tested its effects on reliability and on overall criterion validity. The units of analysis were the algorithms applied to each dataset. For instance, when testing the effects of Parameter 4 on reliability, we considered 378 algorithms x 6 datasets = 2268 units, equally divided between the two levels of the independent variable. For performing these tests, we used a rank-based robust version of the two-way ANOVA [19,30], the independent variables being the parameter under consideration and its interaction with the presence or absence of the built-in penalty in the dataset (from now on, referred to as *built-in factor*). This second factor was included only to investigate potential differences in the effects of the parameters depending on the IAT procedure used (built-in vs. no built-in) and not to test its main effect. We used average ranks of the algorithms as dependent variables and we had the same number of datasets for built-in and no built-in, leading to almost identical ranks between the two options with small variations only due to tied values. Therefore, we did not report the main effect of the built-in factor in the following results section. We used the robust ANOVA as implemented in the R package *asbio* [31]. Significant main effects of each parameter were investigated with robust rank-based, Tukey-type nonparametric contrasts, as implemented in the R package *nparcomp* [32,33]. These robust contrasts are performed by first computing the relative effect size for each group (i.e., the probability an observation randomly chosen from all groups is smaller than a randomly chosen observation from the specific group). Then the differences between each pair of groups in terms of these effect sizes are computed, providing an effect size measure and its respective confidence interval (see [35] for details). We used these contrasts to test whether some options of the parameters increased or decreased the performances significantly compared to other options. Then, in case of interaction effects, we performed the robust contrasts separately for the built-in and no built-in penalty datasets and we performed the test developed by Patel and Hoel [34] and implemented in the R package *WRS* [35]. The Patel and Hoel statistically tests the difference between the probability that a randomly sampled observation of level *i* of factor A is smaller than a randomly sampled observation from another level *j* of factor A at one level of factor B (in this case, built-in) and the same probability at the other level of B (in this case, no built-in). In other words, we used this test to investigate whether the differences between the different options of a parameter are the same for built-in and no built-in. In case of significant difference, we reported the test statistic Δ with confidence intervals around Δ.

The results of the pairwise comparisons for the two types of IAT (built-in and no built-in), as well as for each type of IAT separately, are represented using T-graphs [36]. A T-graph is a simple graphical representation of a series of pairwise comparisons, proposed by [36]. The nodes of the graph represent the levels of the factor, in our case the options of a parameter, the arrows represent their pairwise comparisons. An arrow points from one option to another if the first option outperforms significantly the second. The robust contrasts are transitive [32], therefore if an option X outperforms another option Y and Y outperforms Z, this implies that X outperforms Z. For sake of a clear graphical representation we followed [36] and omitted the direct edges when two nodes could be connected using an indirect path. The complete tables with the exact values of the statistic tests and the effects sizes are reported in the Supporting Information.

## Results

### Effects of each parameter and the built-in factor on validity and reliability

#### Parameter 1: Extreme latencies treatment (see Fig 1)

We performed two 6 (No Treatment vs. Fixed value Trimming vs. Fixed value Winsorizing vs. 10% Statistical Trimming vs. 10% Statistical Winsorizing vs. 10% statistical Inverse Trimming) x 2 (built-in vs. no built-in) robust ANOVAs on overall validity and reliability separately.

**Fig 1:**
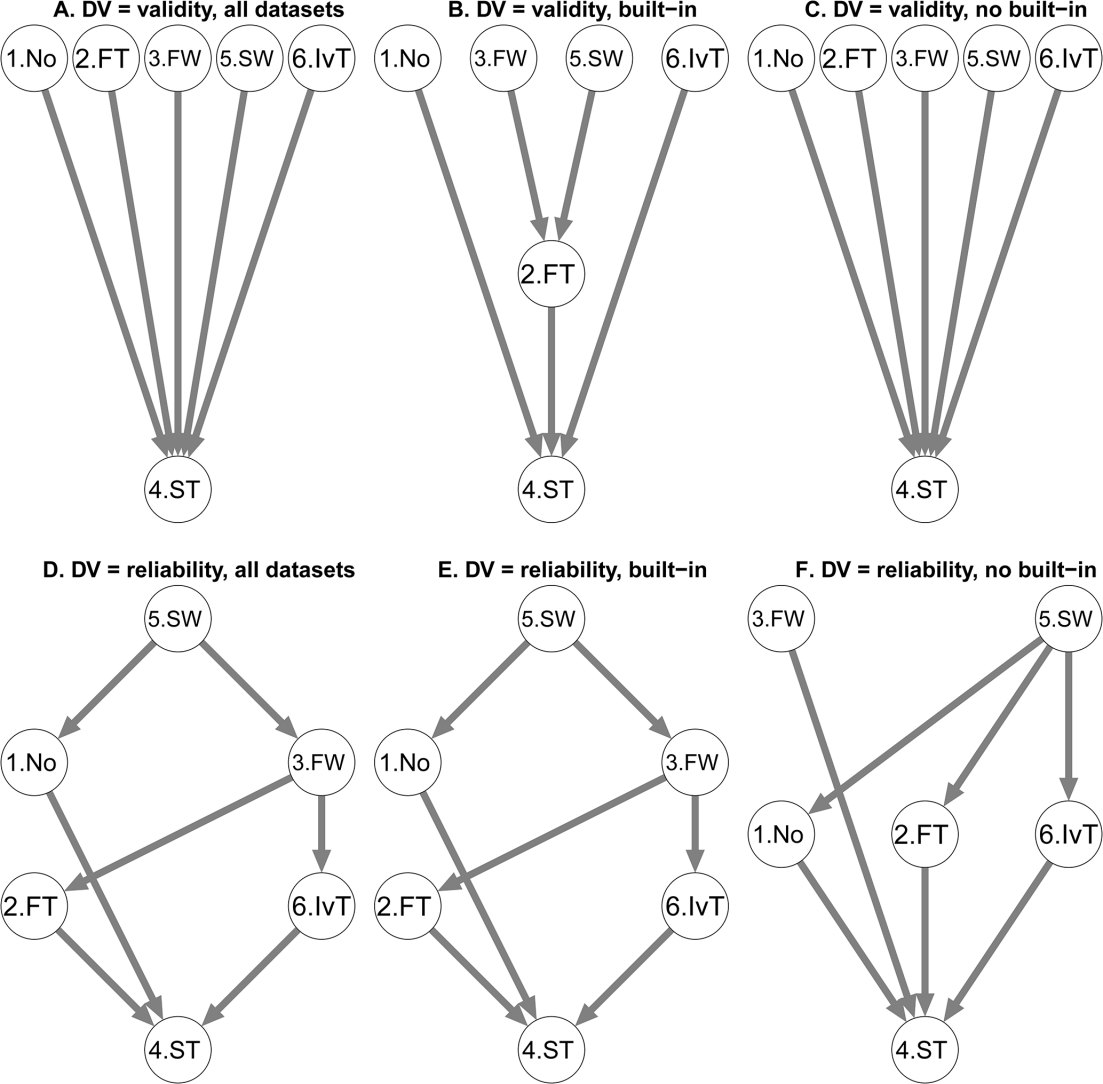
T-graphs for Extreme Correct Latencies Treatment (Parameter 1). Options are coded as follows: 1 (No treatment – No), 2 (fixed value trimming – FT), 3 (fixed value winsorizing—FW), 4 (10% statistical trimming—ST), 5 (10% statistical winsorizing—SW), 6 (10% statistical inverse trimming—IvT). An arrow points from one option to another if the first option outperforms significantly the second. For example, 10% statistical trimming (Node “4. ST”) is outperformed by all other treatments in terms of Validity. Effect sizes are reported in S1 and S2 Tables.

The effect of the type of extreme latencies treatment on validity was significant, *F*(4.96, 2157.42) = 20.11, *p* < .001, $\eta_{p}^{2}$ = .04. Robust contrasts revealed that all the options significantly outperformed option 4, 10% Statistical Trimming (see Fig 1 panel A) with effect sizes ranging from .14 to .18 (see S1 Table.). The interaction term was significant, *F*(4.96, 2157.42) = 5.79, *p* < .001, $\eta_{p}^{2}$ = .01. We applied the robust contrasts separately to the built-in penalty procedure datasets (Fig 1 panel B, see also S1 Table. Built-in panel) and to the no built-in penalty procedure datasets (Fig 1 panel C, see also S1 Table. No built-in panel) and performed the Patel-Hoel test (see S1 Table. Patel-Hoel panel). The main results are twofold. First, 10% Statistical Trimming was outperformed by all other options for both procedures in a similar extent, with only one exception: It was outperformed by 10% Statistical Winsorizing more strongly for built-in procedure than for no built-in procedure, as indicated by the Patel-Hoel test. Second, Fixed values Winsorizing and Statistical Winsorizing outperformed Fixed values Trimming only in the case of a built-in penalty and not of a no built-in penalty.

The type of extreme latencies treatment significantly affected reliability, *F*(4.68, 1857.09) = 97.18, *p* < .001, $\eta_{p}^{2}$ = .20. Similar to what we observed for validity, 10% Statistical Trimming was outperformed by all the other options (Fig 1 panel D) with effect sizes ranging from .25 to .40 (see S2 Table. Total panel). Moreover, option 5, 10% Statistical Winsorizing, obtained better results than any other options with effect size of outperformance ranging from .08 to .40. Finally, Fixed value Winsorizing showed better reliability performance than Fixed value Trimming and Statistical Inverse Trimming. The effect of Parameter 1 was qualified by an interaction with the built-in factor, *F*(4.68, 1857.09) = 3.83, *p* = .002, $\eta_{p}^{2}$ = .01. The outperformance of 10% Statistical Trimming by the other options occurred in both procedures. Moreover, the superiority of 10% Statistical Winsorizing over Fixed value Winsorizing, of Fixed value Winsorizing over Fixed value Trimming, and of Fixed value Winsorizing over statistical Inverse Trimming was statistically significant for built-in penalty data (Fig 1 panel E, see also S2 Table. Built-in panel) but not for no built-in (Fig 1 panel F, see also S2 Table. No built-in panel). Finally, the outperformance of 10% Statistical Trimming by Fixed value Trimming and by statistical Inverse Trimming was stronger for no built-in data than for built-in, as indicated by the Patel-Hoel test (see S2 Table. Patel-Hoel panel).

In sum, 10% Statistical Trimming appears to be the worse option as it was outperformed by all other options on validity and reliability. Moreover, the 10% Statistical Winsorizing seems to be the most efficient way to deal with extreme latencies considering its good performance on reliability and validity compared to the other options. Although there were some significant interaction with the Built-in penalty factor, the inclusion or not of a built-in penalty in the IAT procedure did not change the main results.

#### Parameter 2: Error Treatment (see Fig 2)

We performed two 5 (Ignore vs. Exclude vs. Recode with 2SD vs. Separate vs. Recode with 600) x 2 (built-in vs. no built-in) robust ANOVAs on overall validity and reliability separately. For the following analyses, the duplicate algorithms were considered both within options 1 (Ignore) and 4 (Separate).

**Fig 2:**
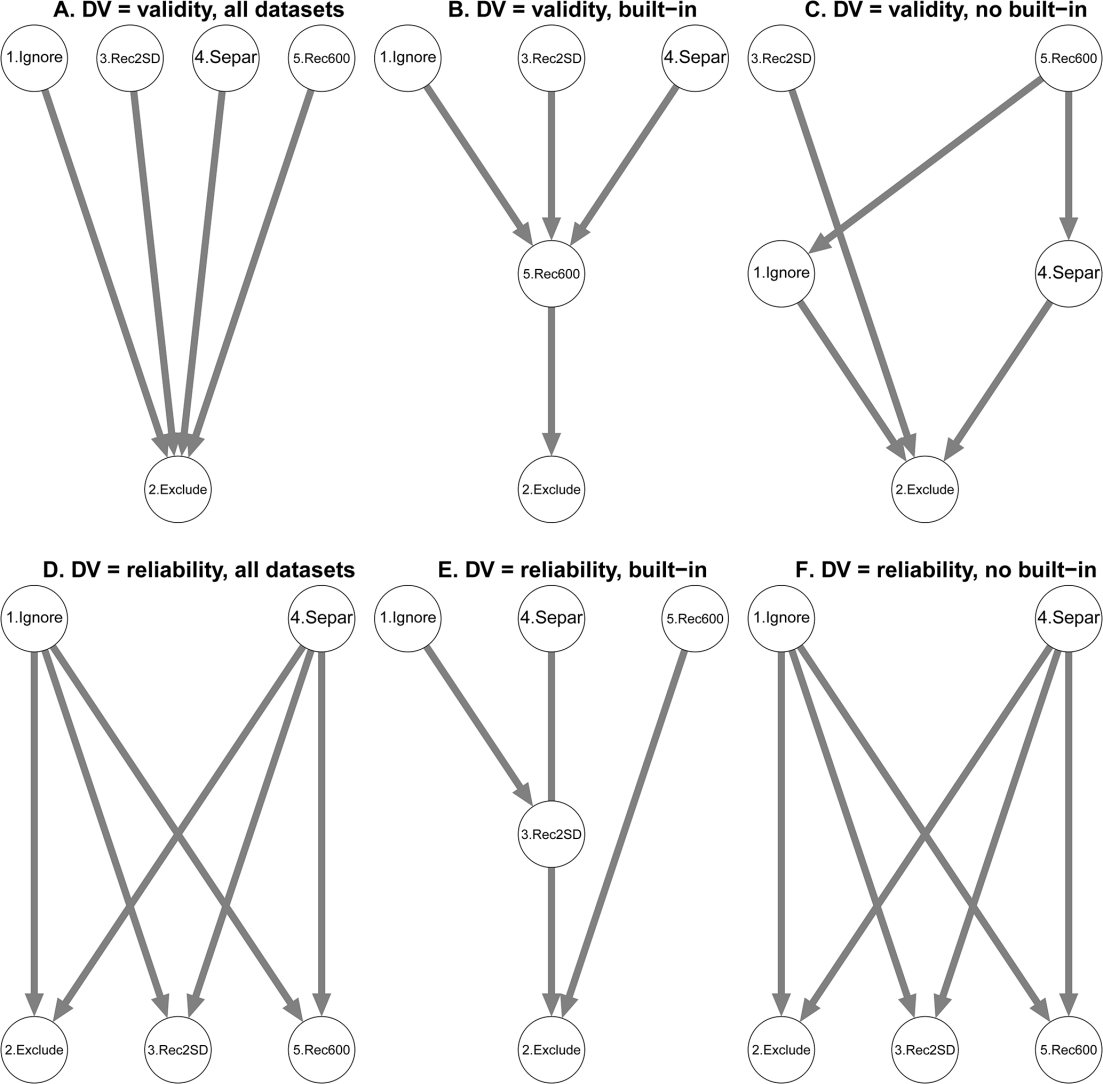
T-graphs for Error Treatment (Parameter 2). Options are coded as follows: 1 (Ignore), 2 (Exclude), 3 (Recode with correct *M* + 2SD – Rec2SD), 4 (Separate – Separ), 5 (Recode 600 with correct *M* + 600 – Rec600). Effect sizes are reported in S3 and S4 Tables.

The type of error treatment on validity was significant, *F*(3.97, 2418.92) = 69.03, *p* < .001, $\eta_{p}^{2}$ = .10. The Exclude option produced worse results than any other methods (see Fig 2 panel A) with effect sizes ranging from .20 to .24 (see S3 Table. Total panel). The effect of Parameter 2 was also qualified by an interaction with the built-in factor, *F*(3.97, 2418.92) = 14.04, *p* < .001, $\eta_{p}^{2}$ = .02. The Patel-Hoel test suggests that outperformance of Exclude by all other options observed with both IAT procedures was always stronger in the built-in penalty than in the no built-in penalty with the exception of the outperformance by the Recode 600 that was not significantly different in the two types of datasets (see S3 Table. Patel-Hoel panel). Moreover, whereas the Ignore and Separate options showed better performance than the Recode 600 in the built-in penalty datasets (see Fig 2 panel B and S3 Table. Built-in panel), this result was reversed with the no built-in data (see Fig 2 panel C and S3 Table. No built-in panel). Finally, the Recode 2SD showed better results than the Recode 600 in the datasets with the built-in penalty whereas there was no difference in case of no built-in penalty (see Fig 2 panels B and C, see also S3 Table. Built-in panel). We also performed a direct comparison of options 1, Ignore, and 4, Separate, after excluding the duplicated algorithms from the data. A robust 2x2 ANOVA revealed a significant main effect, *F*(1, 493.10) = 4.52, *p* = .034, $\eta_{p}^{2}$ = .01, indicating a better performance of the option Separate across IAT procedures, that was not qualified by an interaction effect, *F*(1, 493.10) = 0.03, *p* = .859.

The type of error treatment significantly affected reliability, *F*(3.94, 2410.24) = 22.63, *p* < .001, $\eta_{p}^{2}$ = .04. As shown in Fig 2 panel D, the option Exclude was only worse than Ignore and Separate (effect sizes of .13 and .11, respectively); Ignore and Separate showed better results than both Recode options (effect sizes ranging from .06 to .13) (see S4 Table. Total panel). The main effect was qualified by an interaction with the built-in factor, *F*(3.94, 2410.24) = 7.59, *p* < .001, $\eta_{p}^{2}$ = .01. In the built-in penalty datasets, the option Exclude was worse than all the other options; additionally, option Ignore showed better results compared to Recode 2SD (see Fig 2 panel E, see also S4 Table Built-in panel). In the no built-in penalty, the pattern of results was identical to what we observed on all datasets (see Fig 2 panel F, see also S4 Table. No built-in panel). We also performed a direct comparison of Ignore and Separate, The analysis revealed no significant main effect, *F*(1, 482.99) = 2.81, *p* = .094 and no interaction, *F*(1, 482.99) = 0.44, *p* = .507.

Taken together, the results indicate mainly that excluding errors from the scoring method leads to worse performance in terms of validity and reliability. Again, the inclusion or not of a penalty in the procedure did not affect the main results in an important way.

#### Parameter 3: IAT score formula (see Fig 3)

We performed two 7 (D vs. G vs. Worse Performance Rule vs. Mini Differences vs. 10% statistical Trimming Mini Differences vs. 10% statistical Winsorizing Mini Differences vs. 10% Inverse Trimming Mini Differences) x 2 (built-in vs. no built-in) robust ANOVAs on overall validity and reliability separately.

**Fig 3:**
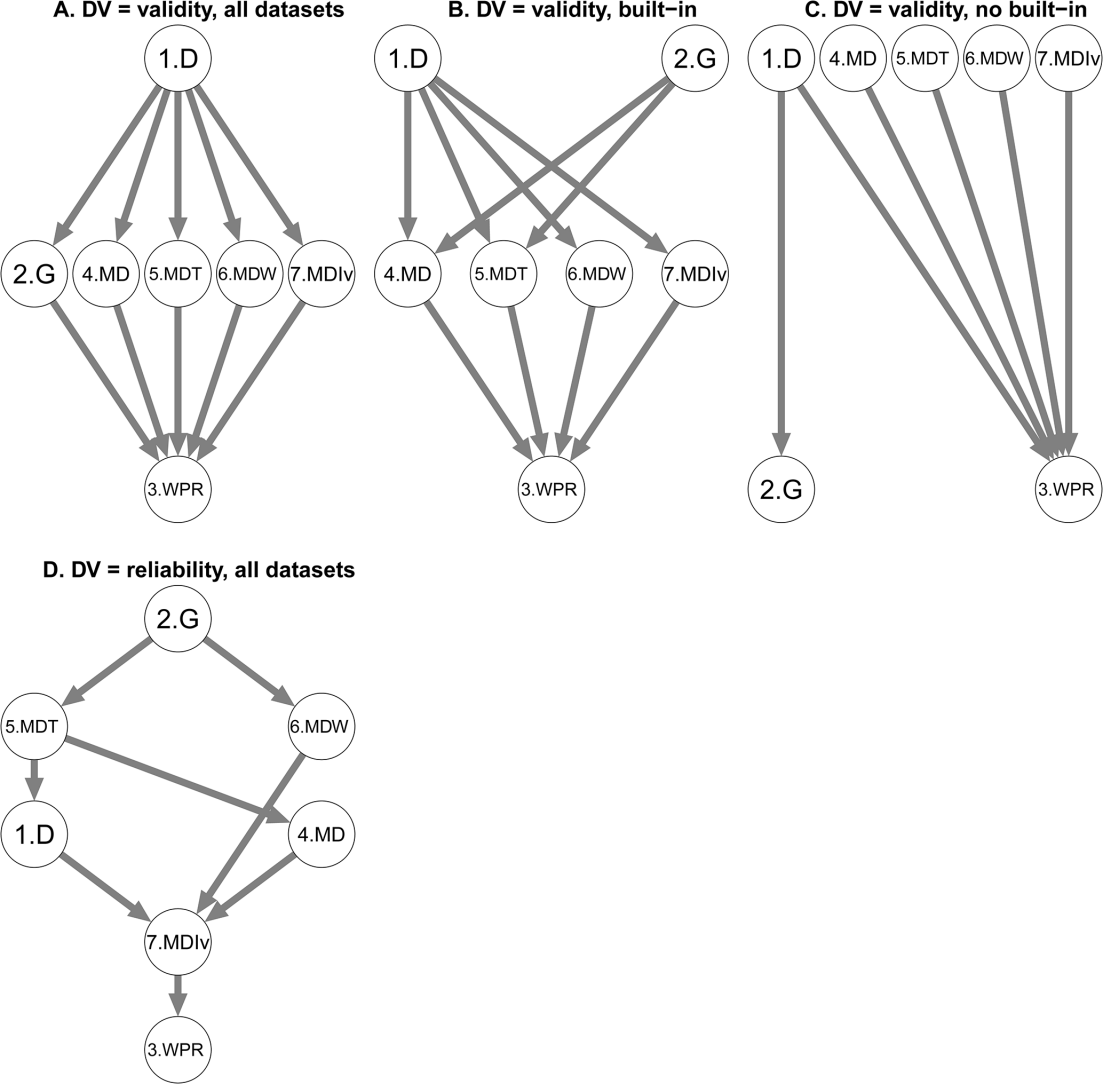
T-graphs for IAT Score Formula (Parameter 3). Options are coded as follows: 1 (D), 2 (G), 3 (Worse Performance Rule—WPR), 4 (Mini Differences—MD), 5 (10% statistical Trimming on Mini Differences—MDT), 6 (10% statistical Winsorizing on Mini Differences—MDW), and 6 (10% statistical Inverse Trimming on Mini differences—MDIvT). Effect sizes are reported in S5 and S6 Tables.

The type of IAT score formula significantly affected validity, *F*(5.74, 2093.12) = 57.42, *p* < .001, $\eta_{p}^{2}$ = .14. The option WPR performed worse than all the other formula and the D was the best option (Fig 3 panel A) with effect sizes ranging from .23 to .35 (see S5 Table. Total panel). The effect of the type of IAT score formula was qualified by an interaction with the built-in factor, *F*(5.74, 2093.12) = 20.99, *p* < .001, $\eta_{p}^{2}$ = .05. In both types of datasets, the WPR always showed worse performances compared to the others but more strongly for built-in than for no built-in data, as indicated by the Patel-Hoel test (see Fig 3 panels A, B, and C and S5 Table.). In the built-in penalty datasets, the D outperformed all other formulas with the exception of the G (see Fig 3 panel B and S5 Table. Built-in panel). In the no built-in penalty datasets, the D scores outperformed the G and the WPR, but not the other formulas. Moreover, the G outperformed WPR, Mini Differences and 10% statistical Trimming Mini Differences in the built-in penalty datasets and not in the no built-in penalty datasets (Fig 3 panels B and C).

The type of IAT score formula also influenced reliability, *F*(5.33, 1891.30) = 234.27, *p* < .001, $\eta_{p}^{2}$ = .40. As illustrated in Fig 3 panel D, the G option outperformed all the other options with effect sizes ranging from .11 to .61 and WPR was outperformed by all the others with effect sizes from .37 to .61. (see S6 Table.). The option 10% statistical Inverse Trimming Mini Differences also performed worse than all other options (effect sizes from .06 to .25) with the exception of WPR. Finally, the option 10% statistical Trimming Mini Differences outperformed the Mini-Differences and the D. There was no interaction with the built-in factor, *F*(5.33, 1891.30) = 0.60, *p* = .713.

In sum, although the G score showed good performance, the D score still appears as a valid option. Moreover, the Worse Performance Rules scoring method leads to the worse results independently from the built-in factor. Once more, the differences between the built-in and no built-in penalty data sets were not important.

#### Parameter 4: Distinction between practice and test critical blocks

We performed two 2 (Distinction vs. No distinction) x 2 (built-in vs. no built-in) robust ANOVAs on overall validity and reliability separately.

The distinction between practice and test critical blocks for computing the IAT score had a significant effect on validity, *F*(1, 2236.85) = 127.51, *p* < .001, $\eta_{p}^{2}$ = .05. The performance of algorithms computed without making distinction performed better than the ones computed taking account such distinction. This effect was qualified by an interaction with the built-in factor, *F*(1, 2236.85) = 95.81, *p* < .001, $\eta_{p}^{2}$ = .04. The Patel-Hoel test revealed that the outperformance of no distinction was stronger for the no built-in penalty procedure datasets than for the built-in, Δ = .22, 95% CI = [.18, .27].

The distinction between practice and test critical blocks also significantly affected reliability, *F*(1, 2123.64) = 66.47, *p* < .001, $\eta_{p}^{2}$ = .03. Like what was observed for validity, the algorithms computed with no distinction performed better compared to the ones with this distinction. There was also a significant interaction with the built-in factor, *F*(1, 2123.64) = 23.88, *p* < .001, $\eta_{p}^{2}$ = .01, with a stronger effect for the no built-in penalty procedure datasets than for the built-in, Patel-Hoel’s Δ = .09, 95% CI = [.04, .14].

#### Performance of the original D scores versus the D scores with some recommended variations

The results from the sub-sections above support some key elements in the D scores as they were originally elaborated [8]. The inclusion of error information and a reduction of the influence of general speed seem to be important elements to obtain satisfactory validity and reliability. However, we believe those D scores can be even more valid when taking into consideration additional results obtained in this contribution. In this perspective, we examined whether the D_2_ for built-in penalty and the D_5_ and D_6_ for no built-in penalty could benefit from the inclusion of two elements that stand out from the results. Within their respective parameter, the Statistical Winsorizing as a treatment for extreme latencies and No distinction between practice and test trials when computing the difference between the two critical blocks seem to lead to the best performances. For each D score, we compared the performance in terms of raw values of the original one with three variants; One computed with no distinction (variant 1), one with Statistical Winsorizing (variant 2), and one with both no distinction and Statistical Winsorizing (variant 3). It appears that the scoring algorithm with both no distinction and Statistical Winsorizing (variant 3) outperforms the original score for the three Ds. For the D_2_, the inclusion of these two variations leads to outperforming the original score 82.9% of the times overall (80% for Validity and 100% for Reliability; see Tables A-C in S2 File). For the D_5_ and the D_6_, it is for both 76.9% of the times overall (70% for Validity and 100% for Reliability) (see Table D in S2 File). In general, the improvements are relatively modest. The improvements in the correlation coefficients are up to .04 for the built-in penalty datasets and .05 for the no built-in penalty datasets. However, considering the type of transformations we applied on the data, one cannot expect huge gains in absolute values. For comparison, when Greenwald et al. (2003, [8] p. 206) examined the effect of different strategies in handling error latencies on the implicit-explicit correlation, the values ranged from approximately *r* = .747 to *r* = .783. Moreover, they recommended the use of the D_2_ over the D_1_ for example, with very modest differences between the performances of the two (from .002 to .003 for implicit and explicit correlation, see Greenwald et al., 2003, [8] Table 2, p. 210). In other words, the differences we obtained between the raw values of the D_2_, D_5_, and D_6_ and those of their improved versions are small yet at least as important as previous improvements on the D score.

## General Discussion

With this contribution, we aimed at testing whether several variations on the scoring algorithm lead to more valid and robust performance of IAT scores. Our approach consisted in the variation of key parameters relevant for IAT data that are the treatment of outliers or extreme latencies, error treatment, method for computing the critical blocks difference, and consideration of practice and test critical block trials. We also included some robust statistic methods theoretically useful for RT data. We used data obtained from the two most frequent IATs format in the literature differing on the inclusion or not of a built-in penalty in the procedure, with 6 datasets of modest and large sample sizes and covering different domains. We ran robust ANOVA on the performances of the algorithms built according to the 4 different parameters. The main results are twofold. First, the results suggest that for most of the parameters we considered some options seem to be better than others. Second, the traditional D scores (D_2_ for built-in penalty IAT procedure, D_5_ and D_6_ for no built-in IAT procedure) show very decent performance although a slight modification such as a statistical winsorizing and no distinction between practice and test critical trials could improve their validity.

### Some recurrent characteristics

Before summing up the main results, discussing some implications, and providing some recommendations on how to handle IAT data, we would like to underline that our contribution does not challenge the results obtained by the traditional D scores. On the contrary, we could consider the results with traditional D scores as an underestimation of what one might obtain by using further improved algorithms, as we demonstrated in the Results section. Although the results of the analyses testing for on each parameters’ main effects do not always allow clearly determining the best option for each parameter, they are relatively informative on some important aspects to consider when elaborating an IAT score that would reflect best an implicit relative preference.

First, the results do not really differ in terms of the IAT procedure. Our aim was not to demonstrate whether one procedure is better than the other but to examine whether the ways to handle errors and reaction time should be similar. Although there were significant interactions between the parameter under examination and the built-in factor, results did not suggest the need for distinct extreme latencies treatment or IAT score formula. Moreover, in both cases, excluding errors seems to be the worst choice. The two sets of data differ on whether the IAT contains a penalty for errors in the procedure. Despite this difference, results are in accordance with the idea that one should integrate errors and latencies in the same index such that errors are recoded through a time penalty. Error latencies seem to contain additional useful information and one should not discard them, whether the IAT includes a penalty or not in its procedure. This overall result does not support Glashouwer et al.’s [12] suggestion that the inclusion versus exclusion of errors does not make a difference. We argue that the IAT performance and thus the implicit relative preference is reflected in part in the mistakes one does when completing the task.

Second, the long tails in reaction times might contain important information but their importance is far from being sufficient. One could argue that because the IAT is supposed to assess preference with a measure based on response interference, the higher tails in the incompatible block and the lower tails in the compatible block might be the only information one might need to elaborate a valid score. However, two of our results advocate against this reasoning. On the one hand, Statistical Trimming leads mostly to the worse results and Fixed Value Trimming does not lead to the best results either, although the latter was recommended by [8]. Instead, 10% Winsorizing is not outperformed by any of the others. In other words, recoding high and low latencies might be better than removing them. On the other hand, the WPR is always outperformed by other options and therefore it is not recommended despite Ratcliff et al.’s [29] suggestion. In sum, although tails might contain important information so that one should recode extreme latencies to reduce the distortion they create without removing the important information they may contain, considering only them is not sufficient.

Third, in accordance with Greenwald et al.’s [8] result, it seems that the best approach is to calculate the difference between the two critical blocks is the D. This method is one way to reduce the effect of the heavy tails of the distributions of each individual affecting both means and *SD*. However, the G also showed satisfactory results with IAT data as it did with BIAT data. In the computation of the G scores one considers the rank of the latencies rather than the raw latencies. This is another way to reduce the influence of outliers in the distribution at the individual level. Although the mini differences also include such a characteristic, they did not receive neither clear support nor clear rejection. Further research may be needed to understand whether it is a viable option. In sum, a method that allows the reduction of the influence of the general response speed is preferable.

Finally, the results clearly indicate that it might be better not to compute the difference between the two critical blocks separately for practice and test trials. Therefore, the idea of giving more weight to practice trials as they might capture better the performance compared to the test trials seems inadequate. It might be true that there is a learning-component in test trials compared to practice, masking the construct-related variance. However, results seem to indicate that this argument is not sufficient to give more weight to practice trials. In sum, although Greenwald et al. [8] suggested this method, our results advocate against it. A possible implication of this result is that it might challenge the need to separate sharply between practice and test critical trials. Future research would be needed to address fully this point.

### Future research

One could wonder why we focused only on the main effects of the parameters and not on their interactions. It is indeed possible to imagine that a certain combination of options of the different parameters yields best performance although the options taken separately would not yield best results. However, a systematic investigation of the interactions among the four parameters plus the built-in factor would have resulted in a 6 × 4 × 7 × 2 × 2 robust ANOVA. Despite the relatively large samples and the use of robust statistics, the results from such a design with our data would not be sufficiently stable and reliable. In fact, in earlier phases of this work, we first examined whether a single algorithm resulting in the combination of different options of different parameters lead to the best performance. The results were not consistent enough to allow firm conclusions and hence we chose the more robust data analytic strategy reported in this manuscript. As we stated in our aims for this contribution, if a certain combination of some transformations improves the psychometric properties of the IAT score, it should be in a consistent manner across samples and domains. Therefore, we preferred to focus on effects we believe are robust, instead of elaborating on interaction effects that might uncover better combinations in our data but that would be perhaps less likely to be replicated. Having said that, future studies with larger samples may refine the strategy by adding interaction effects.

We based all our recommendations on results obtained from robust statistic performed on modest to large and very large sample size datasets. At this level of knowledge, we would also like to invite researchers to consider the specific options we identified in this contribution as possible alternatives to score the IAT. However, despite these strong features, we believe that there is still room for expanding our results upon criteria, domains, characteristics of the participants (e.g., age), and testing conditions (e.g., online vs. lab). This task would be impossible for a single laboratory; therefore, we encourage researchers to replicate our results on different datasets and in different conditions. For this purpose, we implemented an R package [37], *IATscores*, and made it freely available (see S3 File for where to find it and how to use it). The package is meant to facilitate the testing of the effects of the different parameters on new datasets and to lower the barrier to researchers for their use. In this way, results can cumulate over time and future studies could deepen the investigation. We recommend further investigations on specific issues based on the limitations of this research.

First, we stress that the tests of the effects of different ways to handle IAT data should preferably include objective behavioral measures. In the built-in penalty datasets, the web-based aspect of the data collection allowed for large-scale samples but prevented from collecting more observable behavior. In the no built-in penalty datasets, we examined the effects of the parameters with data that included behavioral measures. Further testing of the scoring procedures in terms of predictive validity is essential. Indeed a measure is useful when it predicts the behavioral consequences of the concept that is being measured [3]. Second, for validity, we considered convergent validity with direct and indirect measures, and predictive validity. These properties are usually the most common when testing the validity of a score. However, one could consider other properties such as the ability to discriminate existing groups. We gave the same weight to all psychometric properties related to validity. Some researchers could argue that a scoring method should show good performance on a specific psychometric property and not crucially on others. Depending on the conception of validity, one might want to also consider alternative criteria such as causality (e.g., [38]). For example, researchers might want to test whether an experimental manipulation affects different scores to the same extent. More important, one might hypothesize that depending on whether the IAT is used as a predictor or as a dependent variable, some parameters could be more important. Scoring methods seem to matter when examining the effect of some external factors (e.g., [18,39]). For example, the detection of cognitive load effects mainly depends on the computation method used for the IAT (i.e., log-transformation or individual variability calibration) [18]. In this perspective, the point would be to determine which component such as errors or extreme latencies is important to consider. Even though somehow related to this contribution, the purpose and method would be different. In this case, it would be to evaluate the moderating effect of some variables on the validity of some ways to handle IAT data.

Focusing on the algorithms we tested, we opted for putting aside existing methods for computing the difference between the critical blocks such as the simple mean, the median, or the log-transformed mean. We based our decision on previous results [8,12] showing that these methods never achieved the best results and on the fact that they have been substantially replaced in current use by D-based scores. Moreover, in the case of the BIAT, Nosek et al. [13] showed that a theoretical trimming (i.e., removing latencies above 10000ms and latencies below 400ms) applied to the mean or the log-transformed mean did not produce better results than the D or the G. We do not pretend to have exhausted all parameters and all options to be considered for a valid IAT scoring method. For example, it could be interesting to consider different ways to compute the denominator for reducing the effect of general speed. One might propose an alternative to the inclusive SD and test whether if indeed it leads to better results. Future research could look to this or other aspects that we did not test in this contribution.

Finally, although we tested the algorithms on data from two different IATs, there are other variants such as the personalized IAT without error feedback [40] or the ST-IAT [41]. It would be interesting to see how the different algorithms perform with data from variants of the IAT. More in general, future work should also be oriented toward applying a similar systematic approach to other indirect measures. Some previous work have started to take this approach. Krause et al. [7] pointed out that reliability can be drastically increased by trimming raw latencies before computing APT and ID-EAST scores but they compared the results to a baseline that could be easily improved (split-half reliabilities were .54 for the APT and .34 for the ID-EAST). Bar-Anan and Nosek [23] showed that trimming outlier scores considerably reduced the internal consistency of the AMP as well as its convergence with direct measures. The framework we have used for IAT data could be readily adapted to the specifics of other indirect measures or reaction time paradigms. We argue this line of work should be extended in a more regular way. For example, it could be interesting to investigate whether the same parameters need to be considered for elaborating a score for a measure that relies on response interference and for a measure that does not rely on such principle. As a closing comment, we would like to stress again that research would need to focus on the validity of the scoring procedure (or the validity of a score) as a separable issue from the validity of the measure. This contribution is a step in that direction for the IAT. We hope that researchers will do similar works for other RT paradigms.

To recap, the original D scores are valid, but they can be improved by three simple adjustments: a) do not use fixed value trimming, but rather use fixed value winsorizing; b) do not discard errors, but rather ignore them, or do outlier treatment separately for error and non-error trials; and c) do not compute the difference separately for practice and test trials, but rather pool them together.

## Supporting Information

S1 File Measures and Psychometric properties. Indicators considered for the psychometric properties for the Political dataset (built-in penalty procedure) (**Table A)**. Indicators considered for the psychometric properties for the Race dataset (built-in penalty procedure) **(Table B).** Indicators considered for the psychometric properties for the Self-esteem dataset (built-in penalty procedure) **(Table C).** Indicators considered for the psychometric properties for the Fruit/Snack, Dessert/Fruit, and Morality datasets (no built-in penalty procedure) **(Table D).**
(DOCX)

S2 File Raw values of the original D scores and the modified D scores. Raw values of the original D_2_, and the modified D2s for the Political dataset **(Table A).** Raw values of the original D_2_, and the modified D2s for the Race dataset **(Table B).** Raw values of the original D_2_, and the modified D2s for the Self-esteem dataset **(Table C).** Raw values of the original D_5_, and D_6_ and the modified D_5_s and D_6_s for the three no built-in datasets **(Table D).**
(DOCX)

S3 File How to use the R package for computing different IAT scores. (DOCX)

S1 Table Robust Contrasts for Parameter1 (Treatment of extreme latencies) in the prediction of validity on all the datasets, on built-in penalty, and on no built-in penalty datasets. (DOCX)

S2 Table Robust Contrasts for Parameter1 (Treatment of extreme latencies) in the prediction of reliability on all the datasets, on built-in penalty, and on no built-in penalty datasets. (DOCX)

S3 Table Robust Contrasts for Parameter 2 in the prediction of validity on all the datasets, on built-in penalty, and on no built-in penalty datasets. (DOCX)

S4 Table Robust Contrasts for Parameter 2 in the prediction of reliability on all the datasets, on built-in penalty, and on no built-in penalty datasets. (DOCX)

S5 Table Robust Contrasts for Parameter 3 in the prediction of validity on all the datasets, on built-in penalty datasets, and on no built-in penalty. (DOCX)

S6 Table Robust Contrasts for Parameter 3 in the prediction of reliability on all the datasets. (DOCX)
