# Supplementary material for: Should We Stop Looking for a Better Scoring Algorithm for Handling Implicit Association Test Data? Test of the Role of Errors, Extreme Latencies Treatment, Scoring Formula, and Practice Trials on Reliability and Validity
Source: PLoS One. 2015 Jun 24;10(6):e0129601. doi: 10.1371/journal.pone.0129601 (PMC4481268; doi:10.1371/journal.pone.0129601)
Supplement: S1 File — Indicators considered for the psychometric properties for the Political dataset (built-in penalty procedure) (Table A). Indicators considered for the psychometric properties for the Race dataset (built-in penalty procedure) (Table B). Indicators considered for the psychometric properties for the Self-esteem dataset (built-in penalty procedure) (Table C). Indicators considered for the psychometric properties for the Fruit/Snack, Dessert/Fruit, and Morality datasets (no built-in penalty procedure) (Table D). (DOCX) [file pone.0129601.s001.docx]

**Measures and Psychometric properties**

**Table A. Indicators considered for the psychometric properties for the Political dataset (built-in penalty procedure).**

| Property | | Measure | Code |
| --- | --- | --- | --- |
| Reliability | | - Split-half method with Spearman-Brown correction (*n* = 2907) - Test-retest correlation (*n* = 119) | Splithalf  Testretest |
| Validity | Convergent validity with Direct measures | - Right wing authoritarianism overall score [1] (*n* = 599) - Thermometer difference score (*n* = 597) - Preference for Democrat over Republican item (*n* = 591) - Average explicit items rating (*n* = 675) | rwa  EDemRep  DemRepPrf  eItmDemRep |
|  | Convergent validity with Indirect measures | - Brief IAT [2] (*n* = 360) - Go/No-go Association Task [3] (*n* = 341), - Single-Target Implicit Association Test [4] (*n* = 361) - Sorting Paired Features task [5] (*n* = 375) - Evaluative Priming [6] (*n* = 366) - Affect Misattribution Procedure [7] (*n* = 554) - Speeded Self-response [8] (*n* = 582) | bat  gat  sat  spf  ep  amp  spd |
|  | Predictive validity (Behavioral measures) | - Democrat vote in the last elections (*n* = 313) - Intention to vote a democrat in the next elections (*n* = 563) | Past Vote  Future Vote |

**Table B. Indicators considered for the psychometric properties for the Race dataset (built-in penalty procedure).**

| Property | | Measure | Code |
| --- | --- | --- | --- |
| Reliability | | - Split-half reliability (*n* = 3003) - Test-retest (*n* = 93) | Splithalf  Testretest |
| Validity | Convergent validity with Direct measures | - Modern racism scale [9] (*n* = 670) - Thermometer difference score (*n* = 635) - Preference white over black item (*n* = 620) - Average explicit items rating (*n* = 675) | mrs  ewhtblk  prfWhtBlk  eItmWhtBlk |
|  | Convergent validity with Indirect measures | - Brief IAT [2] (*n* = 356) - Go/No-go Association Task [3]( *n* = 385) - Single-Target Implicit Association Test [4] (*n* = 387) - Sorting Paired Features task [5] ( *n* = 394) - Evaluative Priming [6] (*n* = 369) - Affect Misattribution Procedure [7] (*n* = 516), - Speeded Self-response [8] (*n* = 548) | bat  gat  sat  spf  ep  amp  spd |
|  | Predictive validity (Behavioral measures) | - Time spent interacting with black people (*n* = 631) | contact |

**Table C. Indicators considered for the psychometric properties for the Self-esteem dataset (built-in penalty procedure).**

| Reliability | | - Split-half reliability (*n* = 2894) - Test-retest (*n* = 125) | Splithalf  Testretest |
| --- | --- | --- | --- |
| Validity | Convergent validity with direct measures | - The Rosenberg self-esteem scale [10] (*n* = 607) - Thermometer difference score (*n* = 670) - Preference for self over other item (*n* = 669) | rsnbrg  eSlfOtr  prfSlfOtr |
|  | Convergent validity with Indirect measures | - Brief IAT [2] (*n* = 349) - Go/No-go Association Task [3] (*n* = 288), - Single-Target Implicit Association Test [4] (*n* = 372) - Sorting Paired Features task [5] (*n* = 359) - Evaluative Priming [6] (*n* = 383) - Affect Misattribution Procedure [7] (*n* = 501), - Speeded Self-response [8] (*n* = 562) | bat  gat  sat  spf  ep  amp  spd |

**Table D. Indicators considered for the psychometric properties for the Fruit/Snack, Dessert/Fruit, and Morality datasets (no built-in penalty procedure).**

| *Fruit/Snack* | | | *N* = 109 for all measures |  |
| --- | --- | --- | --- | --- |
| Reliability | | | - Split-half reliability | splithalffs |
| Validity | | Convergent validity with direct measures | - Attitudes towards both eating snacks and eating fruits Time 1 | attfs1 |
|  |  | Predictive validity (Behavioral measures) | - Self-reported behaviour - Actual choice for a free snack or fruit to take | srbfs  choice |
| *Dessert/Fruit* | | |  |  |
| Reliability | | | - Split-half reliability (*n* = 104) | splithalfdf |
| Validity | | Convergent validity with direct measures | - Attitudes towards both eating dessert and eating fruits Time 1 (*n* = 104) - Attitudes towards both eating dessert and eating fruits Time 2(*n* = 104) | attdf1  attdf2 |
|  |  | Convergent validity with indirect measures | - Dessert SC-IAT [4] (*n* = 104) | sc-iat |
|  |  | Predictive validity (Behavioral measures) | - Self-reported behaviour (*n* = 53) - Rapid Picture Choice Task (*n* = 104) | srbdf  rpct |
| *Morality* | | | *N* = 111 for all measures |  |
| Reliability | | | - Split-half reliability | splithalfm |
| Validity | Direct measures | | - Honesty-Humility scale of the HEXACO-PI [11] | explhon |
|  | Predictive validity (Behavioral measures) | | - Whether the participant returned the additional undue lottery ticket | ticket |

**References**

1. Altemeyer B (1996). (1996) Spector, The authoritarian. Cambridge, MA: Harvard Press.

2. Sriram N, Greenwald AG (2009) The Brief Implicit Association Test. Exp Psychol 56: 1–40. Available: http://www.ncbi.nlm.nih.gov/pubmed/19439401. Accessed 30 May 2014.

3. Nosek B, Banaji M (2001) The go/no-go association task. Soc Cogn 19: 625–664. Available: http://guilfordjournals.com/doi/abs/10.1521/soco.19.6.625.20886. Accessed 23 September 2013.

4. Karpinski A, Steinman RB (2006) The single category implicit association test as a measure of implicit social cognition. J Pers Soc Psychol 91: 16–32. Available: http://www.ncbi.nlm.nih.gov/pubmed/16834477. Accessed 17 September 2013.

5. Bar-Anan Y, Nosek B, Vianello M (2009) The sorting paired features task: a measure of association strengths. Exp Psychol: 1–27. Available: http://psycnet.apa.org/journals/zea/56/5/329/. Accessed 24 September 2013.

6. Fazio RH, Jackson JR, Dunton BC, Williams CJ (1995) Variability in automatic activation as an unobtrusive measure of racial attitudes: a bona fide pipeline? J Pers Soc Psychol 69: 1013–1027. Available: http://www.ncbi.nlm.nih.gov/pubmed/8531054.

7. Payne BK, Cheng CM, Govorun O, Stewart BD (2005) An inkblot for attitudes: affect misattribution as implicit measurement. J Pers Soc Psychol 89: 277–293. Available: http://www.ncbi.nlm.nih.gov/pubmed/16248714. Accessed 16 September 2013.

8. Ranganath K, Smith C, Nosek B (2008) Distinguishing automatic and controlled components of attitudes from direct and indirect measurement methods. J Exp Soc …. Available: http://www.sciencedirect.com/science/article/pii/S0022103106001934. Accessed 24 September 2013.

9. McConahay JB (1986) Modern racism, ambivalence, and the Modern Racism Scale. In: Dovidio JF, Gaertner SL, editors. Prejudice, discrimination, and racism. San Diego, CA: Academic Press. pp. 91–125.

10. Rosenberg M (1965) Rosenberg Self-Esteem Scale. New York.

11. Lee K, Ashton MC (2004) Psychometric properties of the HEXACO personality inventory. Multivariate Behav Res 39: 329–358. doi:doi: 10.1207/s15327906mbr3902_8.
