# Supplementary material for: Should We Stop Looking for a Better Scoring Algorithm for Handling Implicit Association Test Data? Test of the Role of Errors, Extreme Latencies Treatment, Scoring Formula, and Practice Trials on Reliability and Validity
Source: PLoS One. 2015 Jun 24;10(6):e0129601. doi: 10.1371/journal.pone.0129601 (PMC4481268; doi:10.1371/journal.pone.0129601)
Supplement: S2 File — Raw values of the original D2, and the modified D2s for the Political dataset (Table A). Raw values of the original D2, and the modified D2s for the Race dataset (Table B). Raw values of the original D2, and the modified D2s for the Self-esteem dataset (Table C). Raw values of the original D5, and D6 and the modified D5s and D6s for the three no built-in datasets (Table D). (DOCX) [file pone.0129601.s002.docx]

**Table A. Raw values of the original D_2_, and the modified D2s for the Political dataset.**

| label | P1 | P2 | P3 | P4 | splithalf | testretest | rwa | EDemRep | PrfDemRep | eItmDemRep | bat | gat | sat | spf | ep | amp | spd | Past  Vote | Future  Vote |
| --- | --- | --- | --- | --- | --- | --- | --- | --- | --- | --- | --- | --- | --- | --- | --- | --- | --- | --- | --- |
| D2 | FT | ignore | D | D | .927 | .655 | -.433 | .602 | .638 | .692 | .649 | .698 | 0.619 | .597 | .409 | .451 | .647 | .665 | .516 |
| D2_ND | FT | ignore | D | D | .924 | .673 | -.437 | .608 | .644 | .693 | .665 | .685 | 0.622 | .594 | .403 | .448 | .64 | .674 | .515 |
| D2_SW | SW | ignore | D | D | .942 | .653 | -.435 | .607 | .643 | .698 | .659 | .728 | 0.621 | .604 | .419 | .455 | .653 | .688 | .532 |
| D2_SWND | SW | ignore | D | ND | .943 | .666 | -.44 | .616 | .65 | .70 | .67 | .724 | 0.621 | .601 | .421 | .461 | .649 | .699 | .535 |

*Note*. D = D score IAT formula; ND = No Distinction between practice and test trials when computing the difference between critical blocks; SW = Statistical Winsorizing.

| label | P1 | P2 | P3 | P4 | splithalf | testretest | mrs | ewhtblk | prfWhtBlk | eItmWhtBlk | bat | gat | sat | spf | ep | amp | spd | contact |
| --- | --- | --- | --- | --- | --- | --- | --- | --- | --- | --- | --- | --- | --- | --- | --- | --- | --- | --- |
| D2 | FT | ignore | D | D | .852 | .386 | .289 | .322 | .32 | .209 | .473 | .477 | .329 | .294 | .302 | .27 | .314 | -.141 |
| D2_ND | FT | ignore | D | D | .847 | .383 | .286 | .313 | .315 | .21 | .49 | .486 | .327 | .302 | .325 | .269 | .312 | -.133 |
| D2_SW | SW | ignore | D | D | .891 | .421 | .296 | .327 | .321 | .211 | .482 | .498 | .327 | .286 | .304 | .281 | .322 | -.144 |
| D2_SWND | SW | ignore | D | ND | .894 | .408 | .297 | .323 | .314 | .209 | .499 | .514 | .339 | .294 | .314 | .284 | .321 | -.13 |

**Table B. Raw values of the original D_2_, and the modified D2s for the Race dataset.**

*Note*. D = D score IAT formula; ND = No Distinction between practice and test trials when computing the difference between critical blocks; SW = Statistical Winsorizing.

| label | P1 | P2 | P3 | P4 |  | testretest | rsnbrg | eSlfOtr | prfSlfOtr | bat | gat | sat | spf | ep | amp | spd |
| --- | --- | --- | --- | --- | --- | --- | --- | --- | --- | --- | --- | --- | --- | --- | --- | --- |
|  |  |  |  |  | splithalf |  |  |  |  |  |  |  |  |  |  |  |
| D2 | FT | ignore | D | D | .802 | .267 | .169 | .129 | .106 | .377 | .286 | .211 | .207 | -.002 | .148 | .144 |
| D2_ND | FT | ignore | D | D | .793 | .292 | .166 | .138 | .101 | .385 | .295 | .207 | .222 | .001 | .148 | .132 |
| D2_SW | SW | ignore | D | D | .849 | .26 | .198 | .125 | .106 | .389 | .301 | .203 | .181 | -.006 | .144 | .158 |
| D2_SWND | SW | ignore | D | ND | .85 | .299 | .199 | .138 | .112 | .406 | .317 | .203 | .202 | .01 | .144 | .158 |

**Table C. Raw values of the original D_2_, and the modified D2s for the Self-esteem dataset**

*Note*. D = D score IAT formula; ND = No Distinction between practice and test trials when computing the difference between critical blocks; SW = Statistical Winsorizing.

**Table D. Raw values of the original D_5_, and D_6_ and the modified D_5_s and D_6_s for the three no built-in datasets.**

| label | P1 | P2 | P3 | P4 | splithalffs | attfs | srbfs | choice | splithalfdf | splithalfm | sciat | attdf1 | attdf2 | srbdf | rpct | explhon | ticket |
| --- | --- | --- | --- | --- | --- | --- | --- | --- | --- | --- | --- | --- | --- | --- | --- | --- | --- |
| D5 | FT | Rec2SD | D | D | .891 | .08 | .119 | .243 | .842 | .646 | .049 | .254 | .246 | .387 | .175 | -.09 | -.218 |
| D5_ND | FT | Rec2SD | D | ND | .884 | .082 | .127 | .239 | .86 | .631 | .111 | .257 | .233 | .439 | .171 | -.125 | -.248 |
| D5_SW | SW | Rec2SD | D | D | .907 | .089 | .119 | .238 | .837 | .648 | .038 | .26 | .251 | .406 | .159 | -.083 | -.217 |
| D5_SWND | SW | Rec2SD | D | ND | .924 | .09 | .129 | .224 | .878 | .669 | .115 | .267 | .244 | .444 | .157 | -.137 | -.244 |
| D6 | FT | Rec600 | D | D | .902 | .085 | .129 | .248 | .792 | .691 | .09 | .255 | .244 | .406 | .181 | -.077 | -.214 |
| D6_ND | FT | Rec600 | D | ND | .906 | .09 | .137 | .243 | .832 | .712 | .142 | .264 | .247 | .446 | .162 | -.105 | -.248 |
| D6_SW | SW | Rec600 | D | D | .914 | .095 | .134 | .244 | .758 | .697 | .084 | .264 | .257 | .422 | .165 | -.063 | -.178 |
| D6_SWND | SW | Rec600 | D | ND | .935 | .10 | .146 | .233 | .802 | .766 | .15 | .273 | .265 | .45 | .152 | -.097 | -.205 |

*Note*. D = D score IAT formula; ND = No Distinction between practice and test trials when computing the difference between critical blocks; SW = Statistical Winsorizing.
