# Supplementary material for: Should We Stop Looking for a Better Scoring Algorithm for Handling Implicit Association Test Data? Test of the Role of Errors, Extreme Latencies Treatment, Scoring Formula, and Practice Trials on Reliability and Validity
Source: PLoS One. 2015 Jun 24;10(6):e0129601. doi: 10.1371/journal.pone.0129601 (PMC4481268; doi:10.1371/journal.pone.0129601)
Supplement: S3 File — (DOCX) [file pone.0129601.s003.docx]

## How to use the R package for computing different IAT scores

We are glad to make the R package *IATscores* freely available on CRAN ([http://cran.r-project.org/web/packages/IATscores/index.html](https://webmail.unimib.it/Redirect/618A6AA3/cran.r-project.org/web/packages/IATscores/index.html)). The package allows computing all of the variations of the IAT scores that we considered in this paper, by combining the four parameters described in the Table 1 in the main document. For installing and loading the package, it is sufficient to run the following line of code in the R window.

install.packages("IATscores")

The main function of the package is RobustScores(). It requires the IAT data as input, formatted in a specific way (described below), and the specifications of the four parameters (here called P1, P2, P3, and P4). For instance, if the IAT data have been stored in a data frame called IATdata, the code RobustScores(IATdata, P1 = "fxtrim", P2 = "ignore", P3 = "dscore", P4 = "dist") computes the D_2_ scores, the code RobustScores(IATdata, P1 = "wins10", P2 = "ignore", P3 = "dscore", P4 = "nodist") computes the D_2_ scores with 10% winsorizing instead of fixed values trimming and no distinction between practice and critical combined block. It is also possible to specify several options for each parameter: in this case, all the scores that result from the combinations of the parameters’ options are computed. For instance the code RobustScores(IATdata, P1 = "fxtrim", P2 = "ignore", P3 = c("dscore", "gscore"), P4 = "dist") computes both the D_2_ scores and the corresponding G scores. If no option is specified for one or more parameter, all of their possible options are included in the analyses. For instance the code RobustScores(IATdata) computes all the 420 algorithms resulting from all the combinations of the four parameters. Functions SplitHalf() and TestRetest() require a similar input and allow to compute split-half and test-retest reliability. All functions in the R package have been fully documented: the documentation for each function can be accessed using the help() syntax, such as help(RobustScores), and includes complete details about the input / output of each function.

The input data should have a precise formatting (see also Table 1 below). One row by trial and includes the following five variables: a) subject (participant ID), b) correct status of the response (1 for correct, 0 for error), c) latency (in ms), d) blockcode ("pair1" for trials of the first critical block, "pair2" for trials of the second critical block), and e) praccrit ("prac" for practice trials of the critical blocks and "crit" for test trials of the critical blocks). A function, Pretreatment(), has been included for helping the user in formatting the data in the appropriate way. We have documented fully all functions in the R package. You can find more details about the correct R syntax specifications in the package help files.

Table 1.
*Example of how IAT data should be formatted to be used as input for* RobustScores()

| subject | correct | latency | blockcode | praccrit |
| --- | --- | --- | --- | --- |
| 1 | 1 | 1341 | pair1 | prac |
| 1 | 1 | 872 | pair1 | prac |
| 1 | 0 | 711 | pair1 | crit |
| 1 | 1 | 948 | pair1 | crit |
| 1 | 1 | 1329 | pair2 | prac |
| 1 | 1 | 509 | pair2 | prac |
| 1 | 0 | 854 | pair2 | crit |
| 1 | 1 | 731 | pair2 | crit |
| … | … | … | … | … |
