# Supplementary material for: Should We Stop Looking for a Better Scoring Algorithm for Handling Implicit Association Test Data? Test of the Role of Errors, Extreme Latencies Treatment, Scoring Formula, and Practice Trials on Reliability and Validity
Source: PLoS One. 2015 Jun 24;10(6):e0129601. doi: 10.1371/journal.pone.0129601 (PMC4481268; doi:10.1371/journal.pone.0129601)
Supplement: S1 Table — (DOCX) [file pone.0129601.s004.docx]

**Table. Robust Contrasts for Parameter1 (Treatment of extreme latencies) in the prediction of validity on all the datasets, on built-in penalty, and on no built-in penalty datasets.**

|  | TOTAL | | | | BUILT-IN | | | | NO BUILT-IN | | | | Patel-Hoel Δ  [95% CI] |
| --- | --- | --- | --- | --- | --- | --- | --- | --- | --- | --- | --- | --- | --- |
| Contrast | Effect size Estimate | 95% CI | Statistic | *p* | Effect size Estimate | 95% CI | Statistic | *p* | Effect size Estimate | 95% CI | Statistic | *p* |  |
| 1.No-2.FT | .04 | [-.02, .10] | 1.95 | .374 | .08 | [0, .16] | 2.76 | .067 | .01 | [-.08, .09] | 0.18 | >.999 |  |
| 1.No-3.FW | .01 | [-.05, .08] | 0.64 | .988 | -.03 | [-.12, .06] | -0.90 | .946 | .06 | [-.03, .14] | 1.88 | .419 |  |
| 1.No-4.ST | .18 | [.12, -.23] | 8.53 | <.001 | .19 | [.11, .26] | 6.68 | <.001 | .17 | [.08, .25] | 5.57 | <.001 |  |
| 1.No-5.SW | .02 | [-.04, .08] | 0.94 | .937 | -.04 | [-.12, .04] | -1.38 | .736 | .08 | [0, .17] | 2.79 | .062 | -.13 [-.25, 0) |
| 1.No-6.IvT | .04 | [-.02, .10] | 2.01 | .337 | .04 | [-.05, .12] | 1.32 | .774 | .04 | [-.04, .12] | 1.50 | .663 |  |
| 2.FT-3.FW | -.03 | [-.09, .03] | -1.26 | .807 | -.11 | [-.19, .02] | -3.61 | .005 | .05 | [-.03, .14] | 1.73 | .515 | -.17 [.30, .03] |
| 2.FT-4.ST | .14 | [.08, .19] | 6.71 | <.001 | .11 | [.03, .18] | 4.05 | .001 | .16 | [.08, .24] | 5.48 | <.001 |  |
| 2.FT-5.SW | -.02 | [-.08, .04] | -1.05 | .902 | -.12 | [-.20, .04] | -4.32 | <.001 | .08 | [0, .16] | 2.65 | .089 | -.21 [-.33, -.08] |
| 2.FT-6.IvT | .00 | [-.06, .06] | -0.01 | >.999 | -.04 | [.12, .04] | -1.52 | .649 | .04 | [-.04, .12] | 1.34 | .762 |  |
| 3.FW-4.ST | .16 | [.10, .22] | 7.70 | <.001 | .22 | [.14, .29] | 7.43 | <.001 | .11 | [.03, .20] | 3.66 | .004 |  |
| 3.FW-5.SW | .01 | [-.06, .07] | 0.26 | >.999 | -.01 | [-.10, .07] | 0.41 | .999 | .03 | [-.06, .11] | 0.85 | .958 |  |
| 3.FW-6.IvT | .03 | [-.03, .09] | 1.30 | .786 | .07 | [-.02, .15] | 2.22 | .230 | -.02 | [-.10, .07] | -0.51 | .996 |  |
| 4.ST-5.SW | -.16 | [-.21, -.10] | -7.78 | <.001 | -.23 | [-.30, -.16] | -8.45 | <.001 | -.09 | [-.17, -.01] | -2.91 | .044 | -.14 [-.25, -.02] |
| 4.ST-6.IvT | -.14 | [-.19, -.08] | -6.96 | <.001 | -.15 | [-.23, -.08] | -5.64 | <.001 | -.13 | [-.20, -.05] | -4.44 | <.001 |  |
| 5.SW-6.IvT | .02 | [-.04, .08] | 1.08 | .890 | .08 | [0, .16] | 2.82 | .056 | -.04 | [-.12, .04] | -1.44 | .701 | .12 (0, .24] |

*Note*. No = No Extreme Latencies Treatment; FT = Fixed Trimming; FW = Fixed Winsorizing; ST = Statistical Trimming; SW = Statistical Winsorizing; IvT = Inverse Trimming. CI with one parenthesis and 0 indicates that 0 is not included but due to two decimals rounding, the value 0 is reported.
