# Supplementary material for: Should We Stop Looking for a Better Scoring Algorithm for Handling Implicit Association Test Data? Test of the Role of Errors, Extreme Latencies Treatment, Scoring Formula, and Practice Trials on Reliability and Validity
Source: PLoS One. 2015 Jun 24;10(6):e0129601. doi: 10.1371/journal.pone.0129601 (PMC4481268; doi:10.1371/journal.pone.0129601)
Supplement: S2 Table — (DOCX) [file pone.0129601.s005.docx]

**Table. Robust Contrasts for Parameter1 (Treatment of extreme latencies) in the prediction of reliability on all the datasets, on built-in penalty, and on no built-in penalty datasets.**

|  | TOTAL | | | | BUILT-IN | | | | NO BUILT-IN | | | | Patel-Hoel Δ  [95% CI] |
| --- | --- | --- | --- | --- | --- | --- | --- | --- | --- | --- | --- | --- | --- |
| Contrast | Effect size Estimate | 95% CI | Statistic | *p* | Effect size Estimate | 95% CI | Statistic | *p* | Effect size Estimate | 95% CI | Statistic | *p* |  |
| 1.No-2.FT | .01 | [-.04, .07] | 0.71 | .980 | .02 | [.02, -.07] | 0.58 | .992 | .01 | [-.07, .09] | 0.42 | .998 |  |
| 1.No-3.FW | -.05 | [-.11, .01] | -2.51 | .119 | -.07 | [-.07, -.16] | -2.42 | .148 | -.04 | [-.3, .04] | -1.38 | .727 |  |
| 1.No-4.ST | .27 | [.22, .31] | 15.68 | <.001 | .23 | [.23, .29] | 9.06 | <.001 | .31 | [.24, .35] | 13.19 | <.001 |  |
| 1.No-5.SW | -.13 | [-.19, .07] | -6.34 | <.001 | -.17 | [-.09, .17] | -5.91 | <.001 | -.10 | [-.18, .02] | -3.49 | .007 |  |
| 1.No-6.IvT | .01 | [-.05, .06] | 0.43 | .998 | .02 | [.02, -.06] | 0.81 | .964 | <.01 | [-.08, .08] | -0.07 | >.999 |  |
| 2.FT-3.FW | .07 | [.01, .13] | 3.19 | .018 | .09 | [.09, .01] | 2.94 | .040 | .05 | [-.03, .14] | 1.80 | .451 |  |
| 2.FT-4.ST | .25 | [.21, .29] | 14.81 | <.001 | .21 | [.21, .27] | 8.14 | <.001 | .30 | [.23, .34] | 13.00 | <.001 | -.10 [-.21, 0) |
| 2.FT-5.SW | -.14 | [-.20, .09] | -7.03 | <.001 | -.19 | [-.10, .19] | -6.35 | <.001 | -.11 | [-.19, -.03] | -3.94 | .001 |  |
| 2.FT-6.IvT | -.01 | [-.06, .05] | -0.31 | >.999 | .01 | [.01, .09] | 0.19 | >.999 | -.01 | [-.09, .06] | -0.52 | .995 |  |
| 3.FW-4.ST | .32 | [.27, .35] | 17.64 | <.001 | .30 | [.30, .35] | 11.19 | <.001 | .35 | [.28, .39] | 13.95 | <.001 |  |
| 3.FW-5.SW | -.08 | [-.14, .02] | -3.67 | .004 | -.10 | [-.10, -.01] | -3.27 | .015 | -.06 | [-.15, .03] | -2.03 | .317 |  |
| 3.FW-6.IvT | .06 | [.01, .12] | 3.03 | .030 | .10 | [.10, .18] | 3.30 | .013 | .04 | [-.04, .12] | 1.37 | .734 |  |
| 4.ST-5.SW | -.40 | [-.42, .34] | -21.96 | <.001 | -.39 | [-.32, -.39] | -15.33 | <.001 | -.41 | [-.44, -.33] | -15.95 | <.001 |  |
| 4.ST-6.IvT | -.26 | [-.30, -.21] | -16.25 | <.001 | -.20 | [-.20, -.26] | -8.73 | <.001 | -.31 | [-.35, -.25] | -14.26 | <.001 | .11 [0, .21] |
| 5.SW-6.IvT | .14 | [.08, .19] | 7.03 | <.001 | .19 | [.19, .11] | 6.97 | <.001 | .10 | [.02, .18] | 3.56 | .006 |  |

*Note*. No = No Extreme Latencies Treatment; FT = Fixed Trimming; FW = Fixed Winsorizing; ST = Statistical Trimming; SW = Statistical Winsorizing; IvT = Inverse Trimming. CI with one parenthesis and 0 indicates that 0 is not included but due to two decimals rounding, the value 0 is reported.
