# Supplementary material for: Should We Stop Looking for a Better Scoring Algorithm for Handling Implicit Association Test Data? Test of the Role of Errors, Extreme Latencies Treatment, Scoring Formula, and Practice Trials on Reliability and Validity
Source: PLoS One. 2015 Jun 24;10(6):e0129601. doi: 10.1371/journal.pone.0129601 (PMC4481268; doi:10.1371/journal.pone.0129601)
Supplement: S3 Table — (DOCX) [file pone.0129601.s006.docx]

**Table. Robust Contrasts for Parameter 2 in the prediction of validity on all the datasets, on built-in penalty, and on no built-in penalty datasets.**

|  | TOTAL | | | | BUILT-IN | | | | NO BUILT-IN | | | | Patel-Hoel Δ  [95% CI] |
| --- | --- | --- | --- | --- | --- | --- | --- | --- | --- | --- | --- | --- | --- |
| Contrast | Effect size Estimate | 95% CI | Statistic | *p* | Effect size Estimate | 95% CI | Statistic | *p* | Effect size Estimate | 95% CI | Statistic | *p* |  |
| 1.Ignore-2.Exclude | .20 | [.16,.24] | 12.19 | <.001 | .30 | [.24,.35] | 13.39 | <.001 | .11 | [.05,.18] | 4.67 | <.001 | .17 [.08, .27] |
| 1.Ignore-3.Rec2SD | -.03 | [-.08,.02] | -1.83 | .353 | -.01 | [-.08,.06] | -0.35 | .997 | -.05 | [-.12,.02] | -2.10 | .221 |  |
| 1.Ignore-4.Separ | -.03 | [-.08,.02] | -1.44 | .599 | -.03 | [-.09,.04] | -1.00 | .851 | -.03 | [-.09,.04] | -0.99 | .861 |  |
| 1.Ignore-5.Rec600 | -.02 | [-.07,.03] | -0.86 | .909 | .08 | [.01,.15] | -3.14 | .015 | -.10 | [-.17,-.03] | -3.78 | .002 | -.18 [-.28, .07] |
| 2.Exclude-3.Rec2SD | -.24 | [-.27,-.19] | -14.38 | <.001 | -.31 | [-.35,-.25] | -14.04 | <.001 | -.17 | [-.23,-.10] | -6.89 | <.001 | -.13 [-.21, -.04] |
| 2.Exclude-4.Separ | -.23 | [-.27,-.19] | -14.09 | <.001 | -.33 | [-.36,-.27] | -15.60 | <.001 | -.14 | [-.20,-.07] | -5.68 | <.001 | .19 [.10, .28] |
| 2.Exclude-5.Rec600 | -.22 | [-.26,-.18] | -13.26 | <.001 | -.22 | [-.27,-.17] | -10.57 | <.001 | -.21 | [-.27,-.14] | -8.49 | <.001 |  |
| 3.Rec2SD-4.Separ | .01 | [-.04,.06] | 0.41 | .994 | -.02 | [-.08,.05] | -0.66 | .964 | .03 | [-.04,.10] | 1.10 | .805 |  |
| 3.Rec2SD-5.Rec600 | .02 | [-.03,.07] | 0.98 | .865 | .09 | [.02,.16] | 3.57 | .004 | -.05 | [-.11,.03] | -1.75 | .405 | .16 [.05, .26] |
| 4.Separ-5.Rec600 | .01 | [-.04,.06] | 0.58 | .979 | .11 | [.04,.17] | 4.33 | <.001 | -.07 | [-.14,.00] | -2.81 | .041 | .18 [.08, .29] |

*Note*. Rec2SD = Recoding error latencies with Block Mean + 2 *SD*; Separ = Separate; Rec600 = Recoding error latencies with Block Mean + 600.
