# Supplementary material for: Should We Stop Looking for a Better Scoring Algorithm for Handling Implicit Association Test Data? Test of the Role of Errors, Extreme Latencies Treatment, Scoring Formula, and Practice Trials on Reliability and Validity
Source: PLoS One. 2015 Jun 24;10(6):e0129601. doi: 10.1371/journal.pone.0129601 (PMC4481268; doi:10.1371/journal.pone.0129601)
Supplement: S4 Table — (DOCX) [file pone.0129601.s007.docx]

**Table. Robust Contrasts for Parameter 2 in the prediction of reliability on all the datasets, on built-in penalty, and on no built-in penalty datasets.**

|  | TOTAL | | | | BUILT-IN | | | | NO BUILT-IN | | | | Patel-Hoel Δ  [95% CI] |
| --- | --- | --- | --- | --- | --- | --- | --- | --- | --- | --- | --- | --- | --- |
| Contrast | Effect size Estimate | 95% CI | Statistic | *p* | Effect size Estimate | 95% CI | Statistic | *p* | Effect size Estimate | 95% CI | Statistic | *p* |  |
| 1.Ignore-2.Exclude | .13 | [.08, .18] | 7.32 | <.001 | .17 | [.10, .23] | 6.85 | <.001 | .10 | [.03,.17] | 4.03 | .001 |  |
| 1.Ignore-3.Rec2SD | .13 | [.08, .17] | 7.04 | <.001 | .09 | [.02, .16] | 3.54 | .004 | .15 | [.09,.22] | 6.10 | <.001 |  |
| 1.Ignore-4.Separ | .02 | [-.03, .07] | 0.99 | .860 | .03 | [-.04, .10] | 1.21 | .743 | .01 | [-.06,.08] | 0.30 | .998 |  |
| 1.Ignore-5.Rec600 | .08 | [.03, .13] | 4.41 | <.001 | .02 | [-.05, .09] | 0.74 | .946 | .13 | [.06,.20] | 4.97 | .000 | -.11 [-21, 0) |
| 2.Exclude-3.Rec2SD | -.01 | [-.05, .04] | -0.34 | .997 | -.08 | [-.14, -.01] | -3.32 | .009 | .05 | [-.02,.12] | 2.00 | .266 | .13 [.02, .23] |
| 2.Exclude-4.Separ | -.11 | [-.16, -.06] | -6.29 | <.001 | -.13 | [-.20, -.07] | -5.56 | <.001 | -.10 | [-16,-.03] | -3.73 | .002 |  |
| 2.Exclude-5.Rec600 | -.05 | [-.10, 0] | -2.69 | .057 | -.15 | [-.21, -.08] | -5.83 | <.001 | .03 | [-.04,.10] | 1.00 | .855 | .17 [.06, .28] |
| 3.Rec2SD-4.Separ | -.11 | [-.15, .06] | -6.00 | <.001 | -.06 | [-.13, .01] | -2.28 | .151 | -.14 | [-.21,-.08] | -5.79 | <.001 |  |
| 3.Rec2SD-5.Rec600 | -.04 | [-.09, .01] | -2.38 | .122 | -.07 | [-.14, 0] | -2.67 | .059 | -.02 | [-.09,.04] | -0.95 | .877 |  |
| 4.Separ-5.Rec600 | .06 | [.01, .11] | 3.43 | .006 | -.01 | [-.09, .06] | -0.44 | .992 | .12 | [.05,.19] | 4.67 | <.001 | -.13 [-.24, .02] |

*Note*. Rec2SD = Recoding error latencies with Block Mean + 2 *SD*; Separ = Separate; Rec600 = Recoding error latencies with Block Mean + 600. CI with one parenthesis and 0 indicates that 0 is not included but due to two decimals rounding, the value 0 is reported.
