# Supplementary material for: Should We Stop Looking for a Better Scoring Algorithm for Handling Implicit Association Test Data? Test of the Role of Errors, Extreme Latencies Treatment, Scoring Formula, and Practice Trials on Reliability and Validity
Source: PLoS One. 2015 Jun 24;10(6):e0129601. doi: 10.1371/journal.pone.0129601 (PMC4481268; doi:10.1371/journal.pone.0129601)
Supplement: S5 Table — (DOCX) [file pone.0129601.s008.docx]

**Table. Robust Contrasts for Parameter 3 in the prediction of validity on all the datasets, on built-in penalty datasets, and on no built-in penalty.**

|  | TOTAL | | | | BUILT-IN | | | | NO BUILT-IN | | | | Patel-Hoel Δ  [95% CI] |
| --- | --- | --- | --- | --- | --- | --- | --- | --- | --- | --- | --- | --- | --- |
| Contrast | Effect size Estimate | 95% CI | Statistic | *p* | Effect size Estimate | 95% CI | Statistic | *p* | Effect size Estimate | 95% CI | Statistic | *p* |  |
| 1.D-2.G | .07 | [.01, .13] | 3.23 | .022 | .05 | [-.03, .13] | 1.86 | .484 | .11 | [.02, .19] | 3.73 | .004 |  |
| 1.D-3.WPR | .35 | [.28, .39] | 16.00 | <.001 | .53 | [.45, .53] | 23.38 | <.001 | .18 | [.09, .26] | 5.84 | <.001 | .31 [.21, .41] |
| 1.D-4.MD | .09 | [.03, .15] | 4.38 | <.001 | .14 | [.06, .21] | 5.18 | <.001 | .05 | [-.04, .14] | 1.72 | .603 |  |
| 1.D-5.MDT | .13 | [.06, .19] | 5.84 | <.001 | .19 | [.11, .26] | 7.00 | <.001 | .08 | [-.01, .17] | 2.59 | .131 |  |
| 1.D-6.MDW | .08 | [.02, .14] | 3.80 | .003 | .11 | [.04, .19] | 4.33 | <.001 | .05 | [-.04, .14] | 1.81 | .544 |  |
| 1.D-7.MDIv | .08 | [.02, .14] | 4.03 | .001 | .12 | [.04, .19] | 4.57 | <.001 | .05 | [-.04, .14] | 1.69 | .624 |  |
| 2.G-3.WPR | .28 | [.22, .33] | 12.82 | <.001 | .48 | [.40, .49] | 19.59 | <.001 | .07 | [-.02, .16] | 2.46 | .178 | .37 [.26, .48] |
| 2.G-4.MD | .02 | [-.04, .09] | 1.05 | .942 | .08 | (0, .16] | 2.96 | .047 | -.05 | [-.14, .03] | -1.87 | .503 | .14 (0, .28] |
| 2.G-5.MDT | .06 | [.01, .12] | 2.56 | .140 | .13 | [.05, .21] | 4.70 | <.001 | -.03 | [-.11, .06] | -0.89 | .973 | .15 [.02, .29] |
| 2.G-6.MDW | .01 | [-.05, .08] | 0.55 | .998 | .06 | [-.02, .14] | 2.18 | .288 | -.05 | [-.14, .04] | -1.71 | .607 |  |
| 2.G-7.MDIv | .02 | [-.05, .08] | 0.71 | .992 | .07 | [-.02, .15] | 2.40 | .189 | -.06 | [-.14, .03] | -1.91 | .476 |  |
| 3.WPR-4.MD | -.26 | [-.31, -.20] | -12.07 | <.001 | -.40 | [-.43, -.33] | -18.50 | <.001 | -.13 | [-.21, -.04] | -4.09 | .001 | -.31 [-.42, -.20] |
| 3.WPR-5.MDT | -.23 | [-.28, -.16] | -10.38 | <.001 | -.35 | [-.39, -.28] | -16.09 | <.001 | -.10 | [-.19, -.01] | -3.14 | .030 | -.30 [-.42, -.19] |
| 3.WPR-6.MDW | -.27 | [-.32, -.21] | -12.31 | <.001 | -.42 | [-.44, -.35] | -18.96 | <.001 | -.12 | [-.21, -.03] | -3.91 | .002 | -.31 [-.42, -.21] |
| 3.WPR-7.MDIv | -.27 | [-.32, -.20] | -12.38 | <.001 | -.41 | [-.44, -.34] | -19.01 | <.001 | -.13 | [-.21, -.04] | -4.13 | .001 | -.31 [-.42, -.21] |
| 4.MD-5.MDT | .03 | [-.03, .10] | 1.56 | .705 | .05 | [-.03, .13] | 1.92 | .445 | .03 | [-.06, .12] | 0.89 | .974 |  |
| 4.MD-6.MDW | -.01 | [-.07, .05] | -0.49 | .999 | -.02 | [-.10, .06] | -0.81 | .981 | .00 | [-.09, .10] | 0.12 | >.999 |  |
| 4.MD-7.MDIv | -.01 | [-.07, .06] | -0.34 | >.999 | -.02 | [-.09, .06] | -0.60 | .996 | -.00 | [-.09, .09] | -0.03 | >.999 |  |
| 5.MDT-6.MDW | -.04 | [-.11, .02] | -2.02 | .404 | -.07 | [-.15, .01] | -2.69 | .095 | -.02 | [-.12, .07] | 0.77 | .988 |  |
| 5.MDT-7.MDIv | -.04 | [-.02, .10] | -1.90 | .482 | -.07 | [-.14, .01] | -2.50 | .150 | -.03 | [-.12, .06] | -0.93 | .968 |  |
| 6.MDW-7.MDIv | .01 | [-.06, .07] | 0.16 | >.999 | .01 | [-.07, .08] | 0.21 | >.999 | -.01 | [-.10, .09] | -0.15 | >.999 |  |

*Note*. D = D score IAT formula; G = G score; WPR = Worse Performance Rule; MD = Mini Differences; MDT = Trimmed Mini Differences; MDW = Winsorized Mini Differences; MDIv = Inverse Trimmed Mini Differences. CI with one parenthesis and 0 indicates that 0 is not included but due to two decimals rounding, the value 0 is reported.
