# Supplementary material for: Should We Stop Looking for a Better Scoring Algorithm for Handling Implicit Association Test Data? Test of the Role of Errors, Extreme Latencies Treatment, Scoring Formula, and Practice Trials on Reliability and Validity
Source: PLoS One. 2015 Jun 24;10(6):e0129601. doi: 10.1371/journal.pone.0129601 (PMC4481268; doi:10.1371/journal.pone.0129601)
Supplement: S6 Table — (DOCX) [file pone.0129601.s009.docx]

**Table. Robust Contrasts for Parameter 3 in the prediction of reliability on all the datasets.**

|  | TOTAL | | | |
| --- | --- | --- | --- | --- |
| Contrast | Estimate | 95% CI | Statistic | *p* |
| 1.D-2.G | -.18 | [-.23,-.12] | -9.14 | <.001 |
| 1.D-3.WPR | .44 | [.38,.44] | 28.78 | <.001 |
| 1.D-4.MD | .01 | [-.04,.07] | 0.56 | .997 |
| 1.D-5.MDT | -.07 | [-.12,-.01] | -3.46 | .009 |
| 1.D-6.MDW | -.03 | [-.09,.02] | -1.72 | .558 |
| 1.D-7.MDIv | .07 | [.02,.12] | 3.74 | .003 |
| 2.G-3.WPR | .61 | [.52,.57] | 34.60 | <.001 |
| 2.G-4.MD | .19 | [.13,.24] | 9.85 | <.001 |
| 2.G-5.MDT | .11 | [.05,.17] | 5.64 | <.001 |
| 2.G-6.MDW | .15 | [.09,.20] | 7.50 | <.001 |
| 2.G-7.MDIv | .25 | [.19,.29] | 12.92 | <.001 |
| 3.WPR-4.MD | -.43 | [-.43,-.37] | -29.13 | <.001 |
| 3.WPR-5.MDT | -.50 | [-.49,-.43] | -30.92 | <.001 |
| 3.WPR-6.MDW | -.47 | [-.47,-.41] | -30.35 | <.001 |
| 3.WPR-7.MDIv | .37 | [.32,.38] | 26.19 | <.001 |
| 4.MD-5.MDT | .08 | [.02,.13] | 4.08 | .001 |
| 4.MD-6.MDW | -.04 | [-.10,.01] | -2.32 | .212 |
| 4.MD-7.MDIv | .06 | [.01,.11] | 3.23 | .020 |
| 5.MDT-6.MDW | .04 | [-.02,.09] | 1.78 | .518 |
| 5.MDT-7.MDIv | .14 | [.08,.19] | 7.19 | <.001 |
| 6.MDW-7.MDIv | .10 | [.05,.16] | 5.50 | <.001 |

*Note*. D = D score IAT formula; G = G score; WPR = Worse Performance Rule; MD = Mini Differences; MDT = Trimmed Mini Differences; MDW = Winsorized Mini Differences; MDIv = Inverse Trimmed Mini Differences.
